# Kanglaite sensitizes colorectal cancer cells to Taxol via NF-κΒ inhibition and connexin 43 upregulation

**DOI:** 10.1038/s41598-017-01480-2

**Published:** 2017-04-28

**Authors:** Yijia Wang, Chunze Zhang, Shiwu Zhang, Zhenying Zhao, Jiawen Wang, Jiali Song, Yue Wang, Jun Liu, Shaobin Hou

**Affiliations:** 1 Tianjin Union Medical Center, Tianjin, 300121 China; 2 Advanced Studies in Genomics, Proteomics, and Bioinformatics, University of Hawaii at Manoa 2538 McCarthy Mall, Snyder Hall, Honolulu, HI 96822 USA

## Abstract

Taxol, a first-line anti-tumour drug, has low effectiveness against colorectal cancer. Combination with other agents is an effective strategy to enhance Taxol cytotoxicity. Kanglaite injection is an extract from *Coix lacryma-jobi* seed and is usually combined with other agents to treat cancer. The aim of this study was to investigate the treatment effect of Taxol combined with Kanglaite on colorectal cancer cell lines. Kanglaite pretreatment followed by Taxol treatment was found to show the best synergism among all combination strategies. This combination also resulted in the smallest tumour volume in a Balb/c mice model. Kanglaite inhibited the expression of nuclear factor (NF)-κΒ and upregulated that of connexin 43, both of which sensitized cancer cells to Taxol. Moreover, Kanglaite increased many cellular variations caused by Taxol, including tubulin polymerization, caspase-3 cleavage, and upregulated expression of survivin and cyclin B1. These results suggest that Kanglaite pretreatment may increase the effect of Taxol on colorectal cancer.

## Introduction

Colorectal cancer is one of the most common types of cancer worldwide, and millions of people are diagnosed as having this condition each year^1^. Although resection is the optimal treatment, chemotherapy and radiotherapy are also needed to prevent distant metastasis and local recurrence. Taxol is used as a first-line chemotherapeutic agent for the treatment of ovarian and breast cancer^2^, but Taxol treatment of colorectal cancer is not effective in clinical chemotherapy. Some potential mechanisms contributing to Taxol resistance include mutation of tubulin^3^ and cellular total antioxidant capacity^4^, but other effects need further investigation. However, combination with other agents is an effective strategy to enhance Taxol cytotoxicity^5^.

In recent years, it has been confirmed that various Chinese herbs have anti-tumour activity. Many of them have been used in combination with chemotherapeutic agents in the treatment of various cancers to reduce the side effects and enhance the efficacy of these agents^6, 7^. Kanglaite (KLT) injection is an extract from *Coix lacryma-jobi* (adlay) seed whose main active ingredient is a triglyceride containing four types of fatty acids. KLT has diphasic broad-spectrum anti-tumour activity^8^. It enhanced efficacy and reduced side effects in the treatment of many cancers, such as gastric cancer^8^, hepatocellular carcinoma^9^, and non-small cell lung carcinoma (NSCLC)^9^, when it was combined with some chemotherapeutic agents. Coix seed extract can regulate the expression of some apoptotic proteins, including wild type p53^10^, bcl-2^10^, Fas^11^, and caspase-3^12^. It is also known to inhibit nuclear factor (NF)-κΒ-dependent transcription^13, 14^, which is recognized as a target for cancer therapy^15^, and there is a particular interest in the possible use of NF-κΒ inhibitors to enhance the effect of other chemotherapeutic drugs via increased apoptosis^16^. The molecular mechanism of Taxol cytotoxicity has been confirmed. Briefly, Taxol binds to the N-terminal region of β-tubulin and stabilizes microtubules. Eventually, the effect results in G_2_/M arrest, which promotes apoptosis^17^. However, administration of lower doses of Taxol activates various survival signals and leads to chemoresistance. It has been confirmed that Taxol activates NF-κΒ which has critical roles in regulating cell survival, proliferation, invasion and metastasis^18^. Some agents were used to inhibit NF-κΒ to sensitize cancer cells to Taxol, such as curcumin^19^ and 1′ S-1′-acetoxyeugenol acetate^20^. Because KLT is an NF-κΒ inhibitor and has anti-tumour activity, we hypothesized that KLT may enhance Taxol cytotoxicity against colorectal cancer cells. In this work, four colorectal cancer cell lines were used to investigate the treatment effect of Taxol combined with KLT.

## Results

### KLT pretreatment enhances the cytotoxicity of Taxol

**Figure 1:**
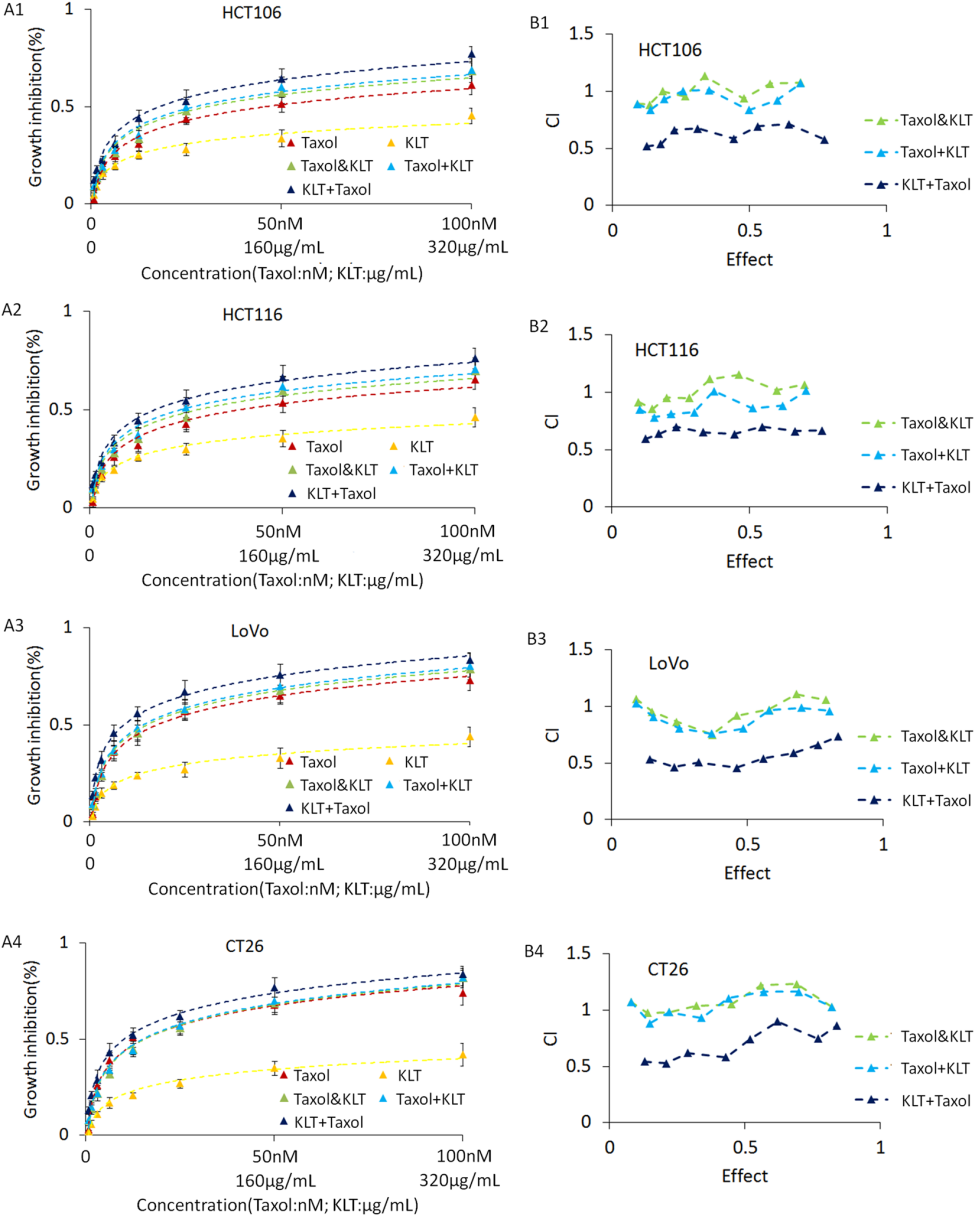
Results of MTT assay and CalcuSyn software analysis for four colorectal cancer cell lines. The groups are defined in “Kanglaite and Taxol treatment”. (**A**) MTT assay results of two agents and three different treatment combinations. Vertical coordinate represents growth inhibition rate, which is compared with that of untreated cells. Horizontal coordinate represents agent concentrations: the first line is Taxol concentration (nM), the second line is KLT concentration (μg/mL). Points represent the mean from three independent experiments and bars represent standard deviations. (**B**) CalcuSyn software analysis of the MTT assay results. “CI” - combination index. CI > 1 represents antagonistic cytotoxicity; CI = 1 represents addictive cytotoxicity; CI < 1 represents synergistic cytotoxicity.

MTT assay showed that Taxol has considerable cytotoxicity against all four cell lines, but KLT has low cytotoxicity against all cells even at high concentrations (>20 μg/ml) (Fig. 1). A higher concentration leads to a change in culture medium components, which may contribute to the inhibition rate increment. Furthermore, KLT enhanced Taxol cytotoxicity when it was administered prior to Taxol treatment, but no obvious synergism was observed when KLT treatment was administered at the same time or after Taxol treatment. Therefore, KLT-pretreated cells were more sensitive to Taxol than untreated cells were, but these cells were not sensitive to KLT itself.

### KLT pretreatment enhances tubulin polymerization induced by Taxol

**Figure 2:**
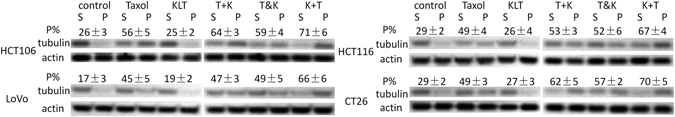
Western blotting analysis of α-tubulin polymerization in four colorectal cancer cell lines: HCT106, HCT116, LoVo, and CT26. Where “t+K” represents “Taxol+KLT”, “t & K” represents “Taxol&KLT” and “K+t” represents “KLT+Taxol”, respectively. Six different drug treatment groups are listed in the top row and are defined in “Kanglaite and Taxol treatment”. “S” represents soluble tubulin and “P” represents polymerized tubulin. Each densitometric value of tubulin was normalized to the densitometric value of β-actin. The percentage of polymerized tubulin was obtained by dividing the densitometric value of the polymerized tubulin by the total tubulin content: $\frac{{P}}{{P}+{S}}$.

The results of both western blotting (Fig. 2) and immunocytochemistry analysis (Supplementary Fig. S1) showed that KLT pretreatment enhanced tubulin polymerization caused by Taxol in all four cell lines. Since Taxol can be taken up by cells and continuously exist, it should be noted that cells assessed at longer times after Taxol treatment have more obvious tubulin polymerization. The “Taxol+KLT” group was observed at an additional 12 h following 12-h Taxol treatment. Taxol existing in these cells still induced tubulin polymerization. For this reason, the group “Taxol+KLT” has a higher tubulin polymerization rate than that of the group “Taxol”. KLT had no effect on tubulin, but when it was combined with Taxol, it increased the tubulin polymerization rate slightly. Furthermore, cells pretreated with KLT and then treated with Taxol had the highest tubulin polymerization rate among all groups.

Immunocytochemistry analyses of α-tubulin (Supplementary Fig. S1) showed that all three human colorectal cancer cell lines in the “KLT+Taxol” group had compact bundles of microtubules and some cells contained asters of microtubules. The cells in the “KLT+Taxol” group also contained many fragmented nuclei stained by DAPI. These results suggest that KLT pretreatment enhanced the cytotoxic effect of Taxol. In the other groups, these three cell lines showed almost the same morphology of microtubules, which may be due to a low concentration of Taxol (6 nM) that cannot induce obvious tubulin polymerization. Furthermore, Taxol caused obvious morphological changes in CT26 cells but changes in microtubule bundles were not obvious. KLT pretreatment also enhanced the morphological changes and increased fragmented nuclei of CT26 cells.

### KLT inhibits NF-κΒ to sensitize colorectal cancer cell lines to Taxol

NF-κΒ is a critical transcription regulator that controls the expression of many genes involved in inflammatory functions, the immune system, and cancer^21, 22^. NF-κΒ is a homodimer or heterodimer composed of two subunits, p50 and p65. The p50-p65 dimer maintains normal physiological function *in vivo*; therefore, we used an anti-p65 antibody to analyse NF-κΒ activation. In normal conditions, NF-κΒ binds to members of the inhibitor of NF-κΒ (IκΒ) family to form an inactivated compound in plasma. When NF-κΒ dissociates from IκΒ upon exposure to an external stimuli, NF-κΒ is activated and transferred to the nuclei. This occurs primarily via activation of IκΒ kinase (IKK). IKK is composed of a heterodimer of IKKα and IKKβ and a regulatory protein (IKKγ). Therefore, we used an anti-IKKα antibody to analyse IKK expression. Furthermore, IKK and IκΒα are both cytoplasmic proteins, so we only detection their expression in cytosolic extraction.

Many anti-tumour drugs, including Taxol, activate NF-κΒ in cancer cells by inducing IκΒ degradation^23^. Studies have shown that Coix seed extract^13^ and KLT^14^ can inhibit NF-κΒ, and cancer cells can be sensitized to Taxol by NF-κΒ inhibition^19^. We investigated whether KLT inhibits NF-κΒ in colorectal cancer cells and sensitizes them to Taxol.

**Figure 3:**
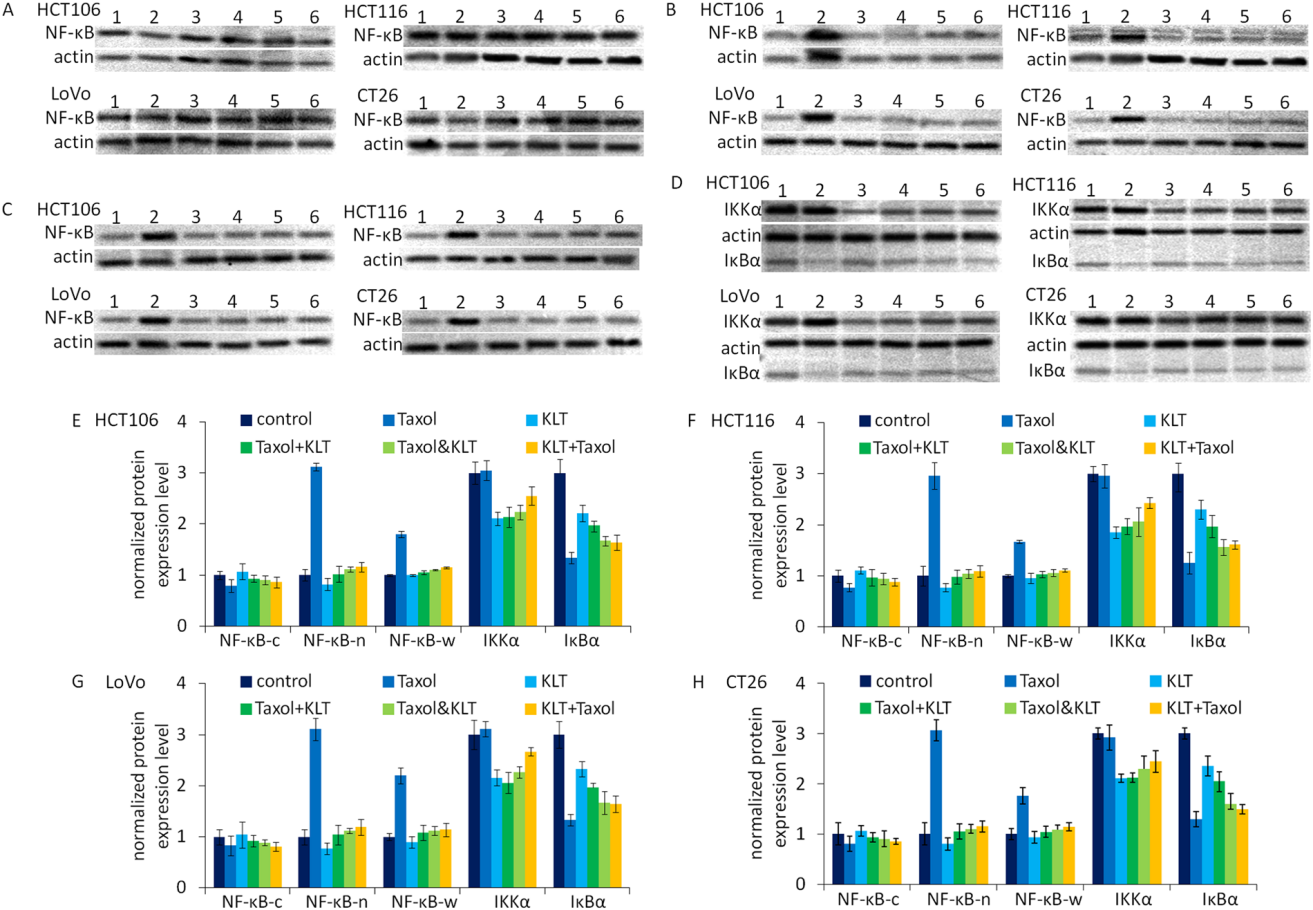
Western blotting analysis of NF-κΒ, IKKα and IκΒα expression in four colorectal cancer cell lines, HCT106, HCT116, LoVo, and CT26, which were treated with different combinations of drugs. β-Actin was used as a loading control. The numbers represent different treatment groups: 1: control; 2: Taxol; 3: KLT; 4: Taxol+KLT; 5: Taxol&KLT; and 6: KLT+Taxol. (**A**) NF-κΒ expression in nuclear; (**B**) NF-κΒ expression in cytoplasm; (**C**) NF-κΒ expression in whole cell lysate; (**D**) IKKα and IκΒα expression in cytoplasm; (**E**–**H**). The densitometric analysis bar diagram of NF-κΒ, IKKα and IκΒα expression in the four colorectal cancer cell lines. Where NF-κΒ-c represents NF-κΒ expression in cytoplasm, NF-κΒ-n represents NF-κΒ expression in nuclear, and NF-κΒ-w represents NF-κΒ expression in whole cell. (**E**) HCT106; (**F**) HCT116; (**G**) LoVo; (**H**) CT26.

The effect of Taxol and KLT on the regulation of NF-κΒ, IκΒα and IKK were determined by western blot. In the cytosolic protein component (Fig. 3A), NF-κΒ was slightly down-regulated by Taxol and slightly upregulated by KLT. Figure 3B showed that Taxol promoted translocation of NF-κΒ to nuclear and KLT can inhibit the translocation effectively. Figure 3C showed that Taxol upregulated NF-κΒ expression in whole cell lysate and KLT can inhibit this effect. Figure 3D showed that Taxol significantly decreased IκΒα expression and KLT also depressed IκΒα expression slightly. Because activation of NF-κΒ can upregulate IκΒ expression to inhibit itself, we speculated that the inhibition of NF-κΒ translocation to nuclear by KLT may decrease IκΒ expression. Figure 3D also showed that KLT but not Taxol decreased IKK expression, which suggested that KLT may inhibit NF-κΒ activation by depressing IKK expression.

Supplementary Fig. S2 also shows that Taxol activated NF-κΒ in all four colorectal cancer cell lines. The red arrows in Fig. S2 indicate that only Taxol group has obvious translocation of NF-κΒ to nuclear but other groups have not. It can be found that KLT pretreatment inhibited NF-κΒ activation induced by Taxol. Treatment combinations of “Taxol+KLT” and “Taxol&KLT” also inhibited NF-κΒ activation, similar to “KLT+Taxol”. Furthermore, the “KLT+Taxol” group showed more fragmented nuclei than the “Taxol” group did, which suggests that KLT inhibits NF-κΒ activation to sensitize cells to Taxol.

### KLT helps Taxol to increase cyclin B1 and survivin expression

**Figure 4:**
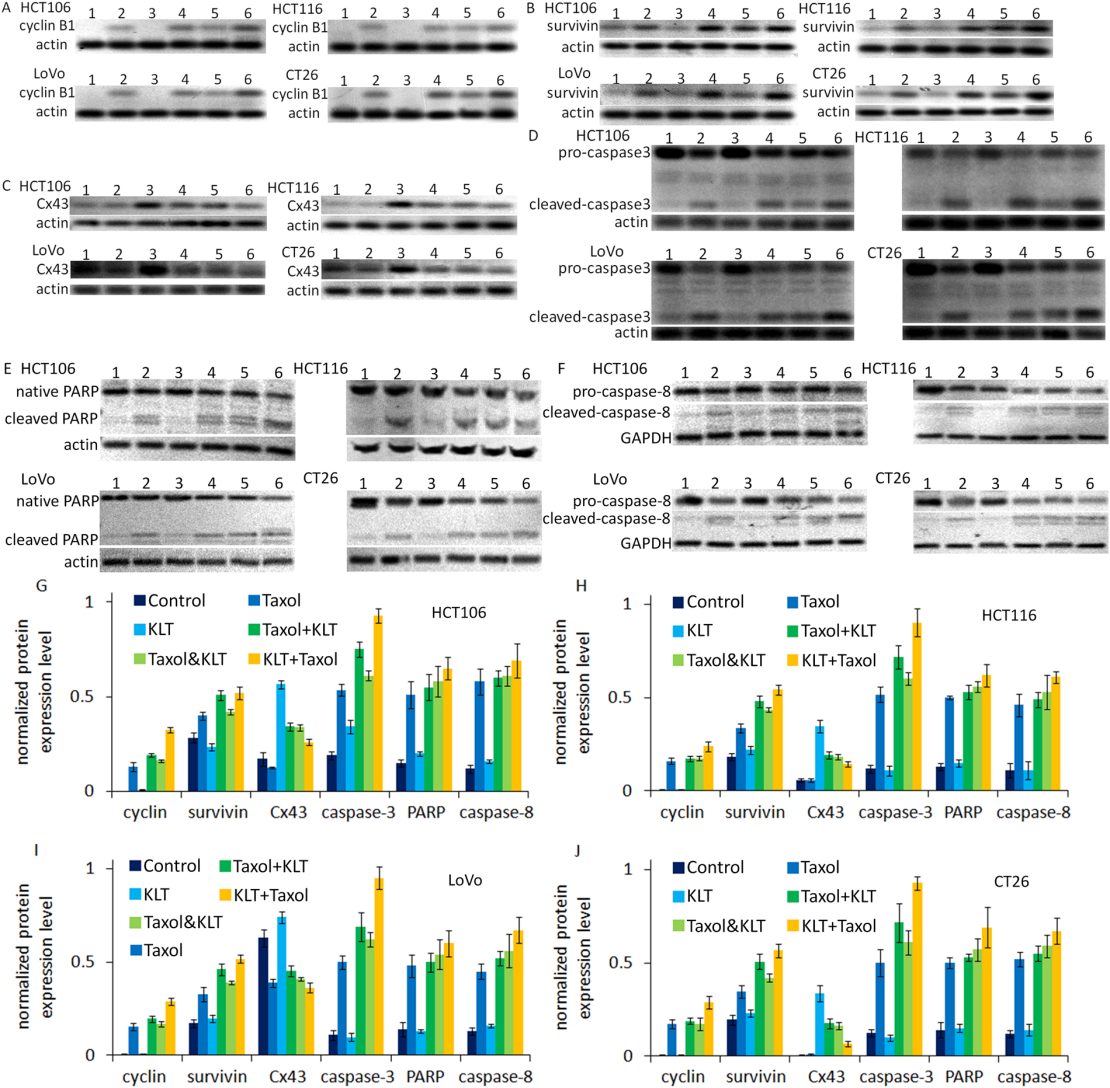
Western blotting results of cyclin B1, survivin, Cx43, caspase-3, PARP and caspase-8 in four colorectal cancer cell lines, HCT106, HCT116, LoVo, and CT26, which were treated with different combinations of drugs. β-Actin or GAPDH was used as a loading control. The numbers represent different treatment groups: 1: control; 2: Taxol; 3: KLT; 4: Taxol+KLT; 5: Taxol&KLT; and 6: KLT+Taxol. (**A**) Cyclin B1 expression in the four cell lines; (**B**) Survivin expression in the four cell lines; (**C**) Cx43 expression in the four cell lines; (**D**) Cleavage of caspase-3 in the four cell lines; (**E**) Cleavage of PARP in the four cell lines; (**F**) Cleavage of caspase-8 in the four cell lines; (**G–J**) The densitometric analysis bar diagram of the results. Columns represent the mean from three independent experiments and bars represent standard deviations. These combinations are defined in “Kanglaite and Taxol treatment”. (**G**) HCT106; (**H**) HCT116; (**I**) LoVo; (**J**) CT26.

Arrest of the cell cycle at the mitotic phase is a molecular mechanism of Taxol-induced cytotoxicity. Taxol causes mitotic arrest at the G_2_/M phase by inducing tubulin polymerization and stabilization. Cyclin B1, a mitotic cyclin, has a crucial role in regulating cyclin-dependent kinase 1, which initiates the progression from G_2_ phase to mitosis. The activity and amount of cyclin B1 increase during the cell cycle until mitosis^24^, after which it degrades^25^. As shown in Fig. 4, the cyclin B1 expression levels were increased by Taxol but not by KLT. In addition, KLT pretreatment resulted in further upregulation of cyclin B1 expression induced by Taxol, and the “KLT+Taxol” group had the highest cyclin B1 expression. Survivin is an anti-apoptotic protein and is highly expressed in various cancers. The survivin expression level increases in the G_2_/M phase of the cell cycle and is always involved in Taxol-mediated mitotic arrest of cancer cells^26^. As shown in Fig. 4, survivin expression levels were increased by Taxol but not by KLT. In addition, KLT pretreatment enhanced the upregulation of survivin expression caused by Taxol, and the “KLT+Taxol” group had the highest survivin expression. These results are consistent with those reported in other studies^27, 28^.

### KLT increases connexin 43 expression, which may increase Taxol cytotoxicity

**Figure 5:**
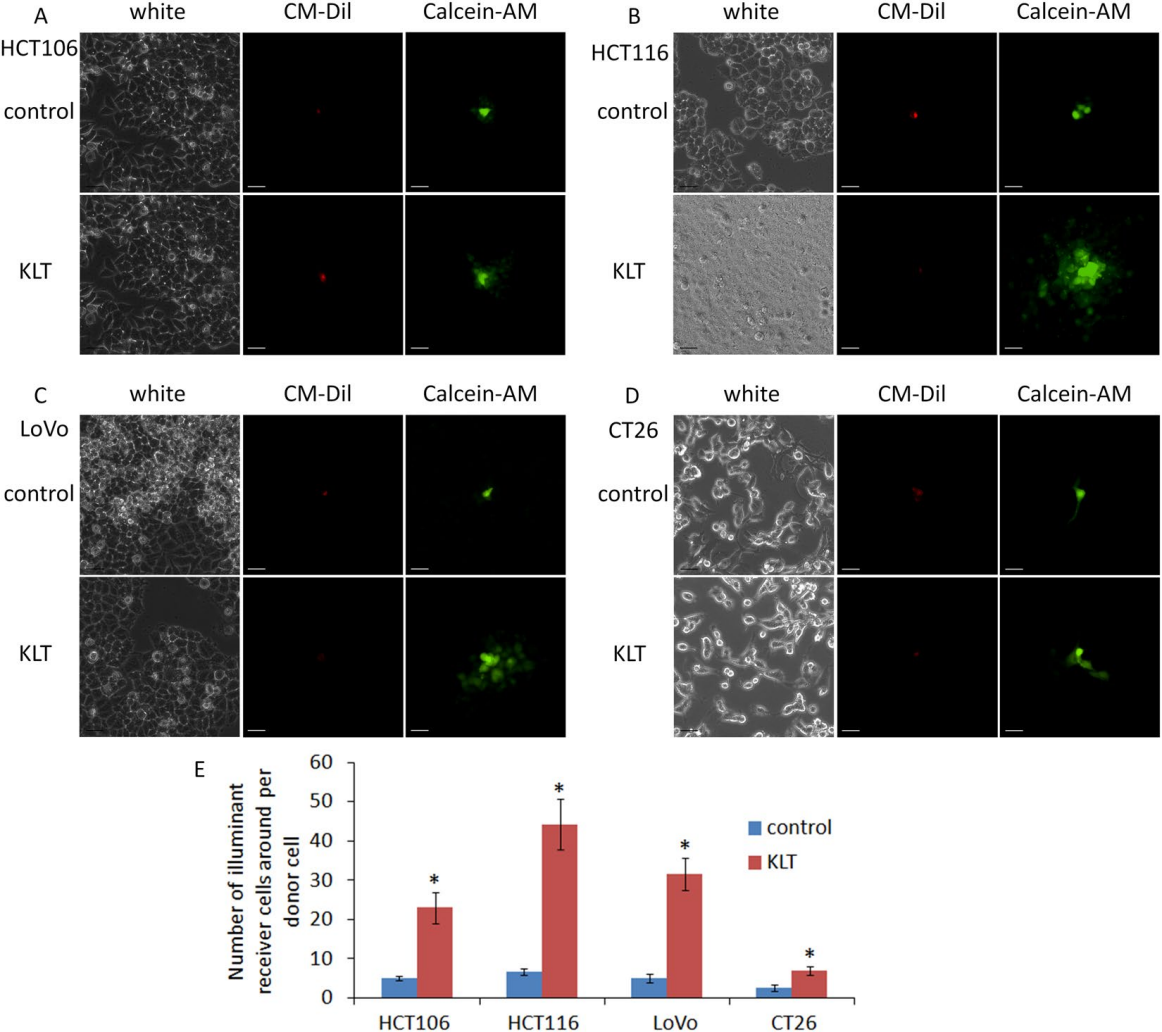
Results of “Parachute” dye-coupling assay. The four cell lines were treated with KLT and GJIC function was measured. The scale bars represent 20 μm. (**A**) HCT106; (**B**) HCT116; (**C**) LoVo; (**D**) CT26; and (**E**) GJIC function in the four cell lines. Columns represent the mean from five independent experiments and bars represent standard deviations. Average number of cells with green fluorescence in each group were compared by student’s t-test. *P < 0.05, significantly different from control group. GJIC functions of all KLT treated cells were higher than control cells.

Connexins are a group of tumour suppressor genes and are potential anti-oncogenic targets for chemotherapy. Upregulating connexin expression can increase the sensitivity of many cancers^29, 30^ to chemotherapy drugs including Taxol^31^. Connexin 43 (Cx43) is the most widely expressed in different tissues among the group of connexins^32^. We found that KLT can upregulate Cx43 expression (Fig. 4), and this effect may enhance the cytotoxicity of Taxol. The “KLT+Taxol” group had lower Cx43 expression than the “KLT&Taxol” and “Taxol+KLT” groups did, which may be because the measurement time was 12 h after KLT treatment. Cx43 is the most ubiquitously expressed member of the connexin family and is a structural component of gap junctional intercellular communication (GJIC), an important intercellular channel^33^. Like Cx43, GJIC function also relates to cellular sensitivity to Taxol^34^. For this reason, we used the “Parachute” dye-coupling assay to measure GJIC function and to determine whether upregulation of Cx43 by KLT treatment can increase GJIC function. Figure 5 shows that KLT-treated cells have higher GJIC function than the control group do. Although upregulation of Cx43 expression only contributes to a part of the GJIC function increment, the results suggest that KLT treatment increases GJIC function by upregulating connexin expression, which may partly relate to Cx43.

### KLT enhances Taxol-induced caspases mediated apoptosis

Caspases are a family of cysteine proteases and are crucial mediators of programmed cell death. It has been confirmed that cleavage of caspases can be induced by Taxol^35^. To further investigate the advantage of the combination of KLT and Taxol, cleavage of caspase-3, caspase-8 and PARP, which are all essential for apoptotic pathways, was analysed by western blotting. Figure 4 shows that Taxol induced cleavage of caspase-3, caspase-8 and PARP, and KLT pretreatment enhanced this effect of Taxol, but KLT cannot induce obvious cleavage of these proteins by itself. Furthermore, the “KLT+Taxol” group showed the highest cleavage level of these proteins.

### “KLT+Taxol” pretreated cells have the lowest tumour growth rate *in vivo*

**Figure 6:**
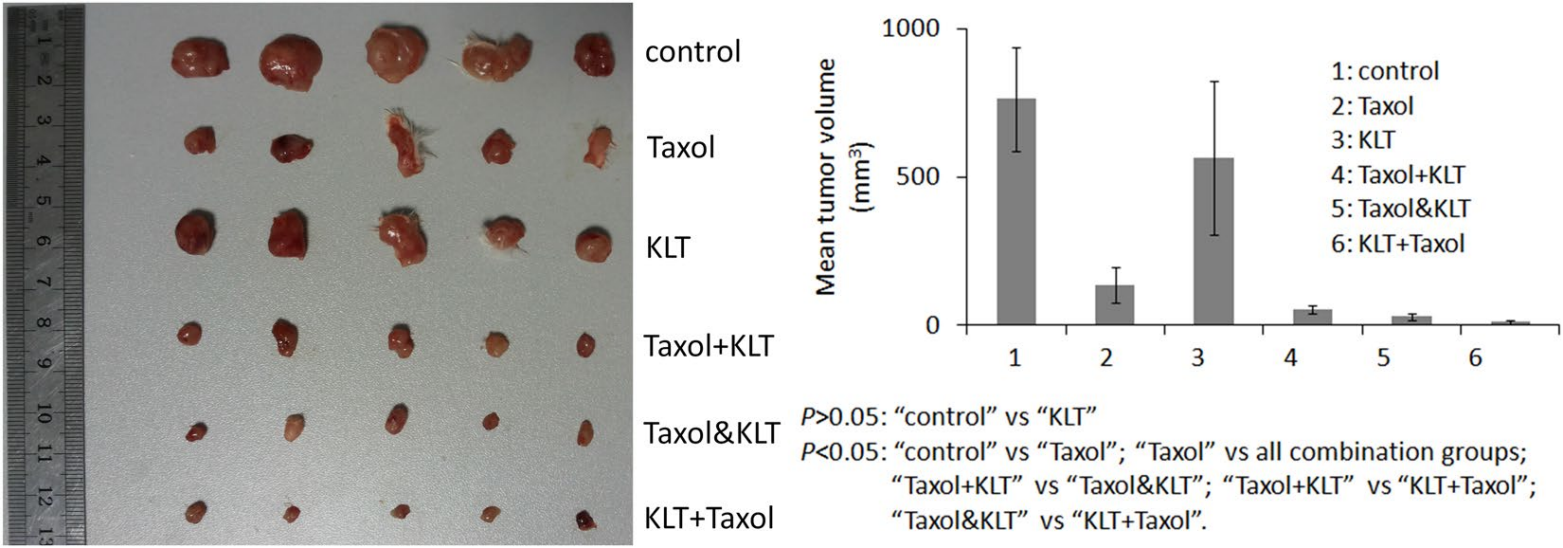
Synergistic inhibition of tumour growth by KLT and Taxol. Five mice were used in each group. Treatment concentrations were 3 nM for Taxol and 10 µg/ml for KLT. Tumours were allowed to develop for 15 days. Columns represent the mean from five tumours and bars represent standard deviations. Average tumour volumes in each group were compared by student’s t-test. *P* < 0.05 represents significant difference.

By comparison with tumours acquired from tumour-bearing mice, it was found that “KLT+Taxol” showed the highest synergism among all combinations (Fig. 6), which is consistent with previous experimental results. Briefly, Taxol-pretreated cells had lower growth ability than the control group cells did, but a single treatment with KLT did not result in a significant inhibition of tumour growth. The combination of “Taxol+KLT” or “Taxol&KLT” pretreatment further decreased tumour volume but these decrements were lower than those reported for “KLT+Taxol”. Furthermore, “Taxol&KLT” had higher synergism than “Taxol+KLT” did, which was different from our western blot and MTT results. This may be because in the previous experiments, cells in the group “Taxol+KLT” were tested 24 h after Taxol was added, but cells in the “Taxol&KLT” group were tested 12 h after Taxol was added. There was no measurement time difference in the mice model, so these results suggested that earlier treatment with KLT resulted in higher synergism than the combination of KLT and Taxol did.

## Discussion

It has been confirmed that KLT has anti-tumour effects when it is combined with chemotherapy. KLT can enhance the short-term efficacy when combined with cisplatin and other agents to treat non-small cell lung cancer (NSCLC)^7^. Furthermore, KLT improved efficacy and reduced the side effects of chemotherapy when it was combined with the DOC regimen, which consisted of docetaxel (D), oxaliplatin (O), and capecitabine (C), to treat gastric cancer^6^. In a recent study, the synergistic mechanism of pemetrexed followed by KLT to treat NSCLC cells was analysed. It was found that *Coix lacryma-jobi* extracts have anti-cancer activities against colorectal cancer cells, but a high concentration was needed. These compounds have IC50s from 35 µg/ml to more than 500 µg/ml for 48 h treatment^36^, or about 100 µg/ml for 24 h treatment^37^. Our results showed that lower anti-proliferative activity may be caused by the shorter treatment time (12 h). However, the mechanism by which KLT enhances chemotherapy efficacy against colorectal cancer remains unclear. For this reason, four colorectal cancer cell lines were analysed *in vitro* and *in vivo*, and our results suggested that the combination “KLT+Taxol” has a higher synergistic effect than “Taxol+KLT” or “Taxol&KLT” do.

Cancer cells often over-express anti-apoptotic proteins to escape apoptosis, so they have a survival advantage^38^. Some conventional anti-tumour agents when used at low concentrations usually upregulate survival signals; therefore, higher concentrations are needed to kill cancer cells, resulting in serious side effects. NF-κΒ is a member of the *Rel* transcription factor family and has critical roles in regulating cell survival, invasion, proliferation, and metastasis. NF-κΒ has a specific intracellular inhibitor named IκΒα, which inactivates NF-κΒ in the cytoplasm. It has been reported that Taxol can downregulate IκΒα to promote the nuclear translocation of activated NF-κΒ^18^. On the contrary, KLT can inhibit NF-κΒ activation^13, 14^, which may be a mechanism by which KLT sensitizes cells to Taxol. Our results were similar among the four colorectal cancer cell lines, indicating the synergistic effect of KLT and Taxol.

The MTT assay results showed that all combinations have higher proliferation inhibition rates than “Taxol” did, but only “KLT+Taxol” showed obvious synergism. This suggests that KLT pretreatment can improve Taxol cytotoxicity significantly but not when used simultaneously or after treatment with Taxol. We found that KLT can upregulate Cx43 expression and GJIC function. GJIC transfers small water-soluble molecules (molecular weight <1 kDa) directly between cells without transporting across the cell membrane^39^; therefore, Taxol or other small molecules involved in apoptosis can be transferred through GJIC. Taxol inhibits GJIC function to counteract its cytotoxicity^39, 40^; therefore, upregulation of GJIC function sensitizes cells to Taxol^41^. Our experiments confirmed that KLT pretreatment increased Cx43 expression and GJIC function, and Taxol treatment can decrease Cx43 expression. The LoVo cell line expresses Cx43^42^, and its GJIC function is also increased by KLT treatment. This effect may contribute to high synergism caused by KLT pretreatment.

Other results of western blot analysis showed that KLT enhances some effects of Taxol, such as promoting caspases related apoptosis, promoting tubulin polymerization, and upregulating cyclin B1 and survivin expression levels. It can be noted that single KLT treatment decreased survivin expression level in HCT106 cells, and increased survivin expression level in other three types of cells, but these variations are small. It is reported that inactivation of NF-κΒ did not affect the activation of survivin because upregulation of survivin by Taxol is regulated by Akt, independent of NF-κΒ^19^, so we speculated that these slight change may due to deviation of western blot experiments.

According to published reports about NF-κΒ inhibition sensitizing cells to Taxol^19, 20^, we suggested that NF-κΒ inhibition caused by KLT is the main reason for synergism observed for the combination treatment in colorectal cancer cell lines. Furthermore, previous studies have shown that long-term pretreatment with a GJIC potentiator can obviously enhance cisplatin cytotoxicity, but this enhanced effect requires a relatively long pretreatment time^29, 43^. Cx43 is a structural component of GJIC which can transfer small water-soluble molecules (molecular weight <1 kDa) directly between cells without passing through cell membrane, so paclitaxel or other small molecules involved in apoptosis can be transferred through GJIC. Therefore, we concluded that upregulation of the Cx43 expression level contributed to “KLT+Taxol” having the highest synergism among all combination groups.

Binding to tubulin and causing tubulin polymerization are the main molecular mechanisms of Taxol cytotoxicity. “KLT+Taxol” increased the polymerization rate and resulted in the highest proliferation inhibition rate among the various combinations. From the tubulin polymerization rate and other indices, it was found that the cytotoxicity of “Taxol+KLT” and “Taxol&KLT” was higher than that reported for the “Taxol” group. KLT pretreatment sensitizes cells to Taxol, so there was still some synergism when KLT and Taxol were used simultaneously. KLT has a low cytotoxicity against colorectal cancer cells, but even though Taxol was removed from culture media, Taxol absorbed in the cells treated with “Taxol+KLT” was continuously effective in the following 12 h before testing. For this reason, “Taxol+KLT” had an even higher effect than “Taxol&KLT”, but the effects of “Taxol+KLT” were lower than “KLT+Taxol”, which further suggests that “KLT+Taxol” has the best synergism.

The cellular experiments required the selection of different drug treatment times for each combination of treatment, but there were no different treatment times in the mice model, so tumour volume may more realistically reflect synergism of different combinations. Comparison of tumour volume showed that “Taxol&KLT” had a greater effect than “Taxol+KLT”, which suggested that the length of KLT treatment is a key influencing factor affecting synergism between KLT and Taxol.

KLT has broad-spectrum anti-tumour activity when it is combined with other chemotherapeutic drugs. The molecular mechanism of KLT and Taxol combination on four colorectal cancer cell lines was investigated in this work. Two single agent groups and three combination groups were analysed, which included “KLT”, “Taxol”, “Taxol+KLT”, “Taxol&KLT”, and “KLT+Taxol”. We found that KLT had little inhibitory effect on colorectal cancer cells but KLT pretreatment sensitized cells to Taxol significantly. KLT inhibited NF-κΒ and upregulated Cx43 expression, which can both enhance Taxol cytotoxicity. KLT pretreatment enhanced some effects of Taxol, such as tubulin polymerization, caspase-3 cleavage, PARP cleavage,caspase-8 cleavage, and increased expression of survivin and cyclin B1. Our results suggest that “KLT+Taxol” has the highest synergism. The Balb/c mice model was also used to verify this conclusion.

## Methods

### Reagents and antibodies

All cell culture media, antibiotics, and trypsin were purchased from Gibco (Grand Island, NY, USA), and foetal bovine serum (FBS) was purchased from HyClone (Logan, UT, USA). Rabbit anti-NF-κΒ p65 antibody, rabbit anti-IKKα antibody, rabbit anti-IκΒα antibody, rabbit anti-caspase-8 antibody, rabbit anti-GAPDH antibody, rabbit anti-Poly[ADP-ribose] Polymerase (PARP) antibody, rabbit anti-cyclin B1 antibody, rabbit anti-survivin antibody, rabbit anti-caspase-3 antibody, mouse anti-α-tubulin antibody, rabbit anti-β-actin antibody, goat anti-rabbit IgG-peroxidase, goat anti-mouse IgG-peroxidase, goat anti-rabbit IgG-FITC, goat anti-mouse IgG-FITC, DAPI, methyl thiazolyl tetrazolium (MTT), and Taxol were purchased from Sigma-Aldrich (St. Louis, MO, USA). Immobilon membranes were purchased from Merck Millipore (Bedford, MA, USA). Cell Tracker CM-Dil and Calcein-AM were purchased from Invitrogen (Carlsbad, CA, USA). ECL Plus substrate, bicinchoninic acid (BCA) reagents, and RIPA lysis buffer were purchased from CWBio (Beijing, China). Kanglaite injection was purchased from Zhejiang Kanglaite Pharmaceutical Co., Ltd, (Zhejiang, China).

### Cell lines

The HCT106, HCT116, LoVo, and CT26 cell lines were purchased from the Shanghai Institutes for Biological Sciences, Chinese Academy of Sciences. HCT106, HCT116, and LoVo cell lines are human colorectal carcinoma cell lines. CT26 are a murine colon adenocarcinoma cell line derived from Balb/c mice treated with N-nitroso-N-methylurethane. All these cells were cultured in RPMI 1640 medium supplemented with 10% FBS, 100 U/mL penicillin, and 100 μg/mL streptomycin.

### Kanglaite and Taxol treatment

All four cell lines were treated with various concentrations of KLT and Taxol, with six different models (Fig. 1): (1) Cells without drug treatment were the control group; (2) Cells treated with Taxol alone for 12 h were named the “Taxol” group; (3) Cells treated with KLT alone for 12 h were named the “KLT” group; (4) Cells treated with a mixture of Taxol and KLT for 12 h were named the “Taxol&KLT” group; (5) Cells treated with Taxol for 12 h, washed with PBS, and then treated with KLT for 12 h were named the “Taxol+KLT” group; and (6) Cells treated with KLT for 12 h, washed with PBS, and then treated with Taxol for 12 h were named the “KLT+Taxol” group. Cell viability was measured by MTT assay. The rate of cell growth inhibition in each well was calculated by defining the absorption of cells not treated with agents (control) as 100%. Measurements were performed in triplicate.

### Western blot analysis

All four cell lines treated using the six different models were used as samples. Taxol (6 nM) and 20 μg/mL KLT were used to treat cells in all of the following sections with the exception of the subcutaneous tumour model. Tubulin polymerization; expression of survivin, cyclin B1, NF-κΒ, IKKα, IκΒα and connexin 43; and cleavage of caspase-3, caspase-8, PARP were determined by western blot analysis.

For tubulin polymerization analysis, polymerized and soluble tubulins of cells were separated using a previously reported method^44^. In brief, 10^6^ harvested cells were lysed with 100 μl of hypotonic buffer (20 mM of Tris-HCl [PH 6.8], 2 mM EGTA, 0.5% NP-40, 1 mM of MgCl_2_, and protease inhibitor mixture [Roche Applied Science]) at 37 °C for 5 minutes in the dark. An additional 100 μl of hypotonic buffer was added to the cell lysate. The cell lysate was briefly vortexed and sonicated on ice. The cell lysate was then centrifuged for 10 minutes at room temperature. Polymerized tubulin was in the pellet fraction and soluble tubulin was in the supernatant fraction. The pellets were resuspended in 200 μl hypotonic buffer.

For detection of NF-κΒ, IKKα and IκΒα expression, cytosolic and nuclear proteins were isolated from harvested cells using a nuclear and cytoplasmic protein extraction kit (Beyotime, Shanghai, China) according to manufacturer’s instructions. Briefly, cytosolic protein extraction agent A (200 μl) with 1 mM PMSF was added to havested cells (20 μl) at 4 °C. The cells were resuspended by vortexing vigorously for 10 s. After 15 min incubation on ice, 10 μl cytosolic protein extraction agent B was added, and the tubes were vortexed vigorously for 5 s. After 1 min incubation on ice, tubes were vortexed vigorously for 5 s, and then centrifuged for 5 min at 15000 g at 4 °C. The supernatant, containing the cytoplasmic fraction, was removed into clean, pre-chilled tubes on ice and stored at −80 °C until use. The insoluble pellet containing the nuclear fraction was resuspended in nuclear protein extraction agent (50 μl) with 1 mM PMSF, vortexed vigorously for 20 s and incubated on ice for 30 min. During this incubation, the tubes were vortexed for 15 s every 5 min for six times in total. The tubes were then centrifuged at 15000 g for 10 min at 4 °C. The supernatant, which contained the nuclear fraction, was transferred to clean, pre-chilled tubes on ice and stored at −80 °C until use.

For detection of other proteins, 10^6^ harvested cells were lysed with 500 ml RIPA lysis buffer containing a protein inhibitor for 20 minutes on ice. The cell lysates were centrifuged and protein content in supernatants was quantitated by bicinchoninic acid reagents. Protein samples were suspended in SDS-loading buffer and boiled. Protein (10 μg) was then run on SDS-PAGE gels and transferred to an Immobilon membrane by a semi-dry blotting method. The membrane was probed with primary and secondary antibodies successively using standard techniques. Finally, the signals were visualized by ECL and the membranes were exposed to film. Each assay was performed in triplicate.

### Immunocytochemistry analysis

Immunofluorescence staining was adapted to analyse tubulin polymerization and NF-κΒ activation, which were both induced by Taxol. In brief, cells were washed gently with PBS to remove culture medium, fixed with 10% formalin at room temperature for 20 minutes, treated with 0.5% Triton X-100 for 5 min at 4 °C, and then blocked with 5% normal goat serum overnight at 4 °C. The slides were washed with PBS and incubated with an anti-α-tubulin antibody for 1 h at 37 °C. After washing with PBS, the slides were incubated with FITC-conjugated antibody for 1 h at 37 °C. Subsequently, the slides were washed with PBS to remove antibody and sealed. The coverslips were photographed immediately using an Olympus fluorescence microscope (Olympus, Japan).

### “Parachute” dye-coupling assay

Functional GJIC was examined by the “Parachute” dye-coupling assay, which was described by Wang *et al*.^28^. Cells were seeded in 6-well plates and grown to 80–100% culture density where GJIC formation was possible. Two fluorescent dyes, CM-Dil and Calcein-AM, were used to detect GJIC function. CM-Dil cannot spread to coupled cells but Calcein-AM can be converted intracellularly to calcein, which is a GJIC-permeable dye. Donor cells of each well were stained with 5 μg/ml CM-Dil and 10 μg/ml Calcein-AM for 30 minutes at 37 °C. Subsequently, donor cells were washed with PBS to remove unincorporated dye and trypsinized to be seeded onto a monolayer of receiver cells grown in another well. The ratio of donor to receiver cells was 1:150. After 4 h incubation at 37 °C, GJICs were formed between donor and receiver cells and were detected by an Olympus fluorescence microscope (Olympus, Japan). Red fluorescence of CM-Dil was used to locate donor cells. Calcein-AM emitted green fluorescence and was used to calculate the average number of illuminant receiver cells around each donor cell. This average number was used to evaluate GJIC function. It should be noted that trypsinized donor cells hardly adhere to the new dish surface if they have been treated by Taxol. Therefore, we only measured the effect of KLT treatment on GJIC function.

### Subcutaneous tumour model

A subcutaneous tumour model was used to evaluate inhibition effect of KLT+Taxol treatment on tumours *in vivo*. To prepare CT26 tumour cells for injection, cells were treated with different drug combinations: “control”, “Taxol”, “KLT”, “Taxol+KLT”, “Taxol&KLT”, and “KLT+Taxol”. In each group, the amount of Taxol used was 3 nM and the amount of KLT used was 10 µg/ml. The treatment time for all groups is the same as that described in “Kanglaite and Taxol treatment”. Treated cells were trypsinized and about 10^6^ cells were subcutaneously inoculated into the flanks of the mice (Balb/c, six-week-old, female). Five mice were used per group. Tumours were allowed to develop for 15 days. Mice were sacrificed, and tumours were removed to compare their volume. Callipers were used to measure tumour size. Tumour volume was estimated as: L × S^2^/2, where “L” represents the larger diameter and “S” represents the smaller diameter. Principles of laboratory animal care were followed and all procedures were conducted according to the guidelines established by the National Institutes of Health, and every effort was made to minimize suffering. This study was approved by the Tianjin Union Medical Center Animal Studies Committee.

## Electronic supplementary material

supplementary information
