# Author Correction: Kanglaite sensitizes colorectal cancer cells to Taxol via NF-κΒ inhibition and connexin 43 upregulation

**DOI:** 10.1038/s41598-018-24089-5

**Published:** 2018-04-12

**Authors:** Yijia Wang, Chunze Zhang, Shiwu Zhang, Zhenying Zhao, Jiawen Wang, Jiali Song, Yue Wang, Jun Liu, Shaobin Hou

**Affiliations:** 1 Tianjin Union Medical Center, Tianjin, 300121 China; 2 Advanced Studies in Genomics, Proteomics, and Bioinformatics, University of Hawaii at Manoa 2538 McCarthy Mall, Snyder Hall, Honolulu, HI 96822 USA

Correction to: *Scientific Reports* 10.1038/s41598-017-01480-2, published online 28 April 2017

This Article contains typographical errors. In the Results section under subheading ‘KLT inhibits NF-κΒ to sensitize colorectal cancer cell lines to Taxol’,

“In the cytosolic protein component (Fig. 3A), NF-κΒ was slightly down-regulated by Taxol and slightly upregulated by KLT.”

should read:

“In the cytoplasmic protein component (Fig. 3A), NF-κΒ was slightly down-regulated by Taxol and slightly upregulated by KLT.”

In the Methods section under subheading ‘“Parachute” dye-coupling assay’,

“Functional GJIC was examined by the “Parachute” dye-coupling assay, which was described by Wang *et al*.^28^”

should read:

“Functional GJIC was examined by the “Parachute” dye-coupling assay, which was described by Yu *et al*.^30^”

