# Supplementary material for: Kanglaite sensitizes colorectal cancer cells to Taxol via NF-κΒ inhibition and connexin 43 upregulation
Source: Sci Rep. 2017 Apr 28;7:1280. doi: 10.1038/s41598-017-01480-2 (PMC5430786; doi:10.1038/s41598-017-01480-2)
Supplement: Supplementary file 1 — supplementary information [file 41598_2017_1480_MOESM1_ESM.pdf]

Kanglaite sensitizes colorectal cancer cells to Taxol via NF-κB inhibition and connexin 43 upregulation

Yijia Wang[1,+], Chunze Zhang[1,+], Shiwu Zhang[1], Zhenying Zhao[1], Jiawen Wang[1], Jiali Song[1], Yue Wang[1], Jun Liu[1,*], Shaobin Hou[2,*]

[1]Tianjin Union Medical Center, Tianjin, 300121, China

[2]Advanced Studies in Genomics, Proteomics, and Bioinformatics, University of Hawaii at Manoa 2538 McCarthy Mall, Snyder Hall, Honolulu, HI 96822, USA

*corresponding author: Jun Liu(junliu_sci@163.com) and Shaobin Hou(shaobin@hawaii.edu)

[+]these authors contributed equally to this work

Supplementary materials

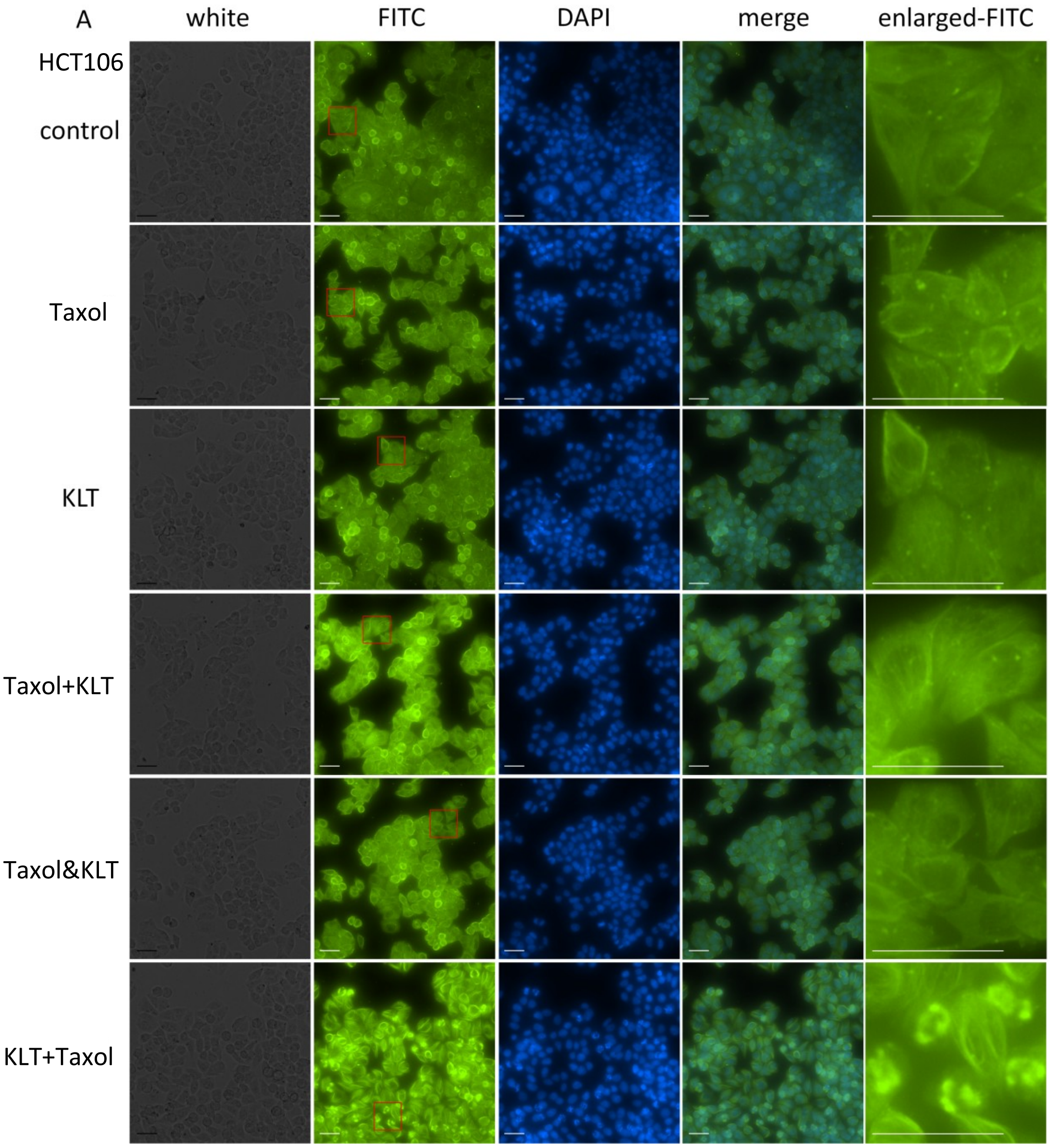

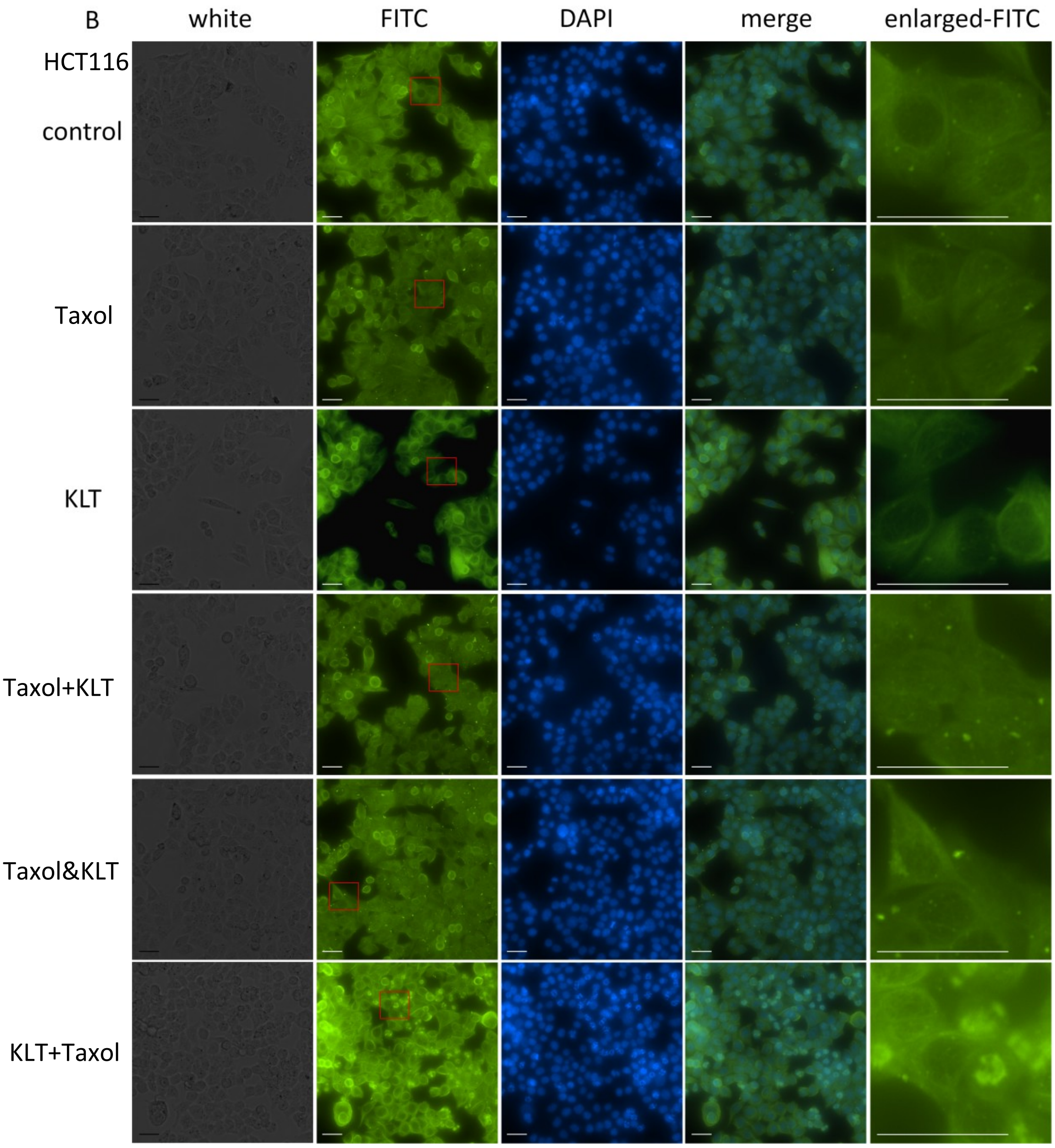
B
white
FITC
DAPI
merge
enlarged-FITC
HCT116
control
Taxol
KLT
Taxol+KLT
Taxol&KLT
KLT+Taxol

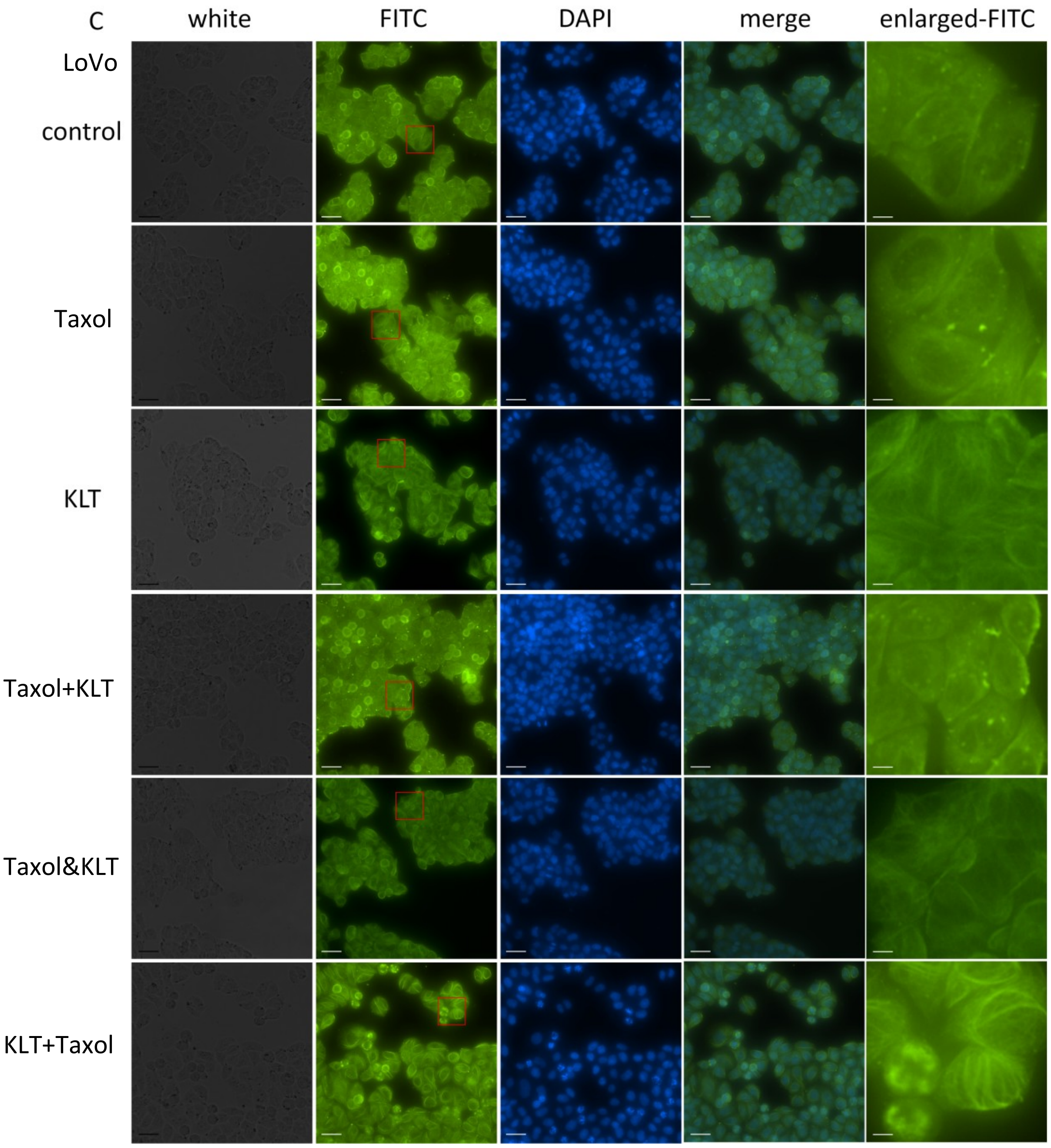
C
white
FITC
DAPI
merge
enlarged-FITC
LoVo
control
Taxol
KLT
Taxol+KLT
Taxol&KLT
KLT+Taxol

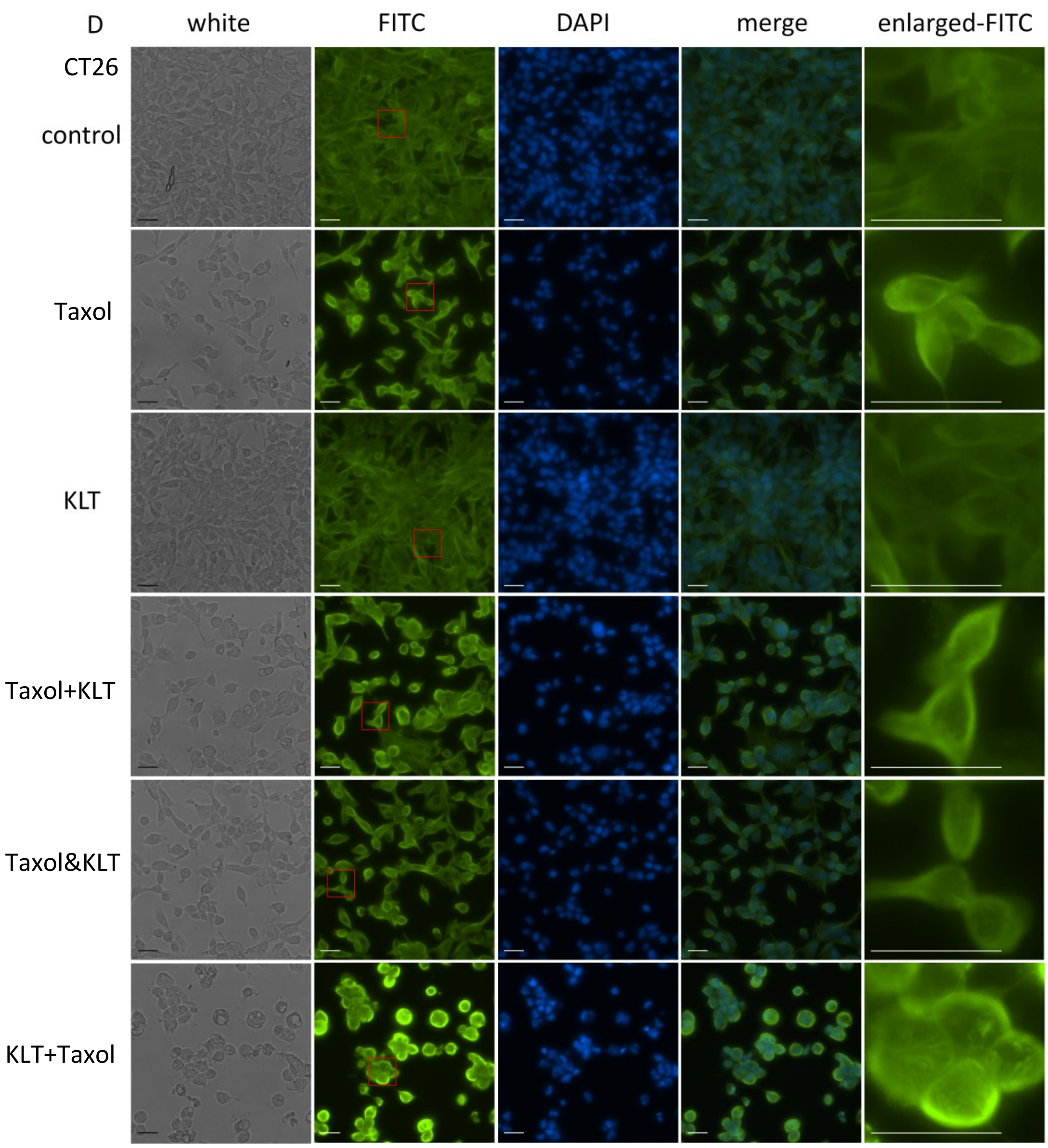

Fig.S1. Fluorescent micrographs of four colorectal cancer cell lines immunostained with α-tubulin antibody followed by FITC-conjugated antibody. Six different drug treatment groups are listed in the left column. These combinations are defined in “Kanglaite and Taxol treatment”. The scale bars represent 20 µm. (A) HCT106; (B) HCT116; (C) LoVo; and (D) CT26.

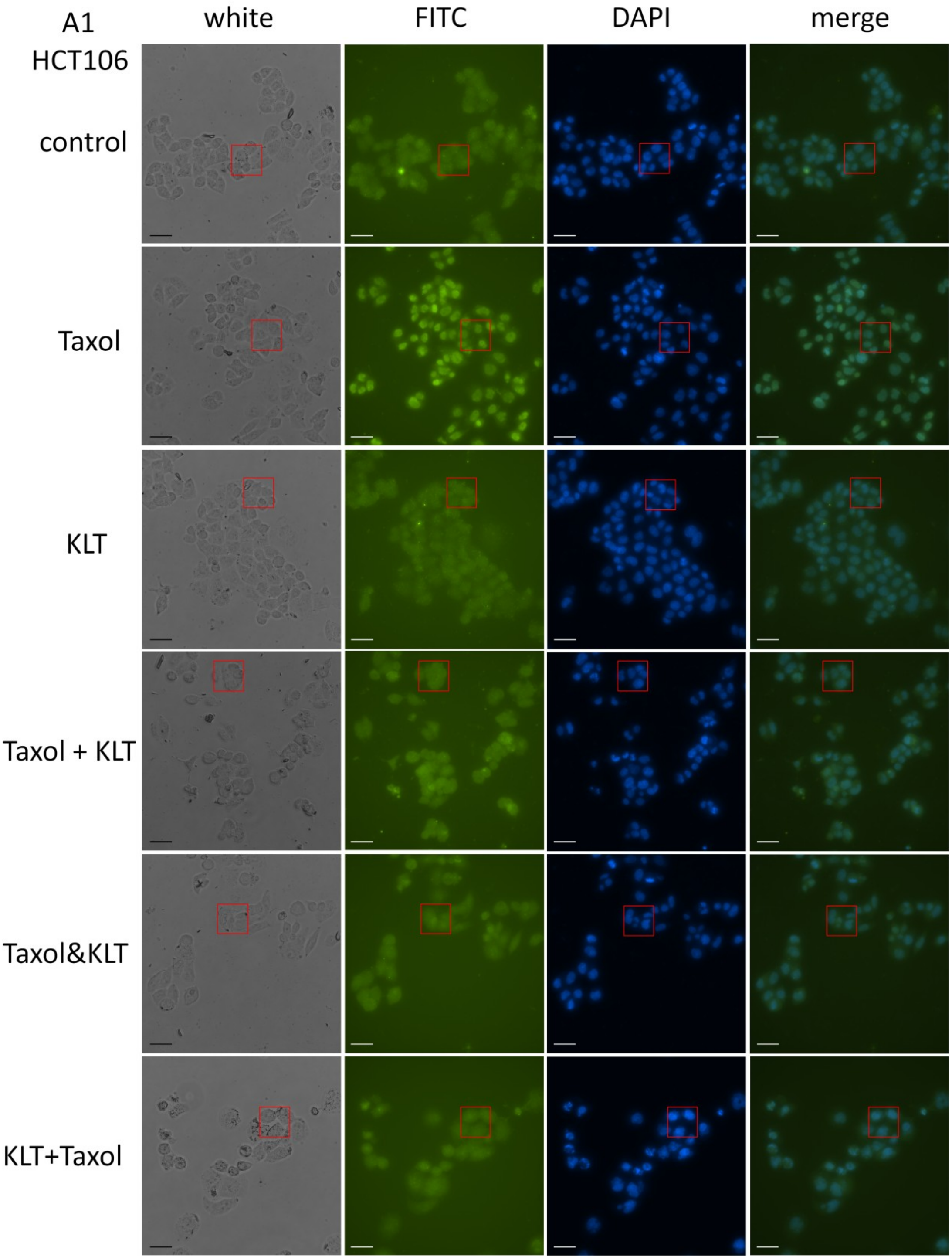

A1
HCT106
white
FITC
DAPI
merge
control
Taxol
KLT
Taxol + KLT
Taxol&KLT
KLT+Taxol

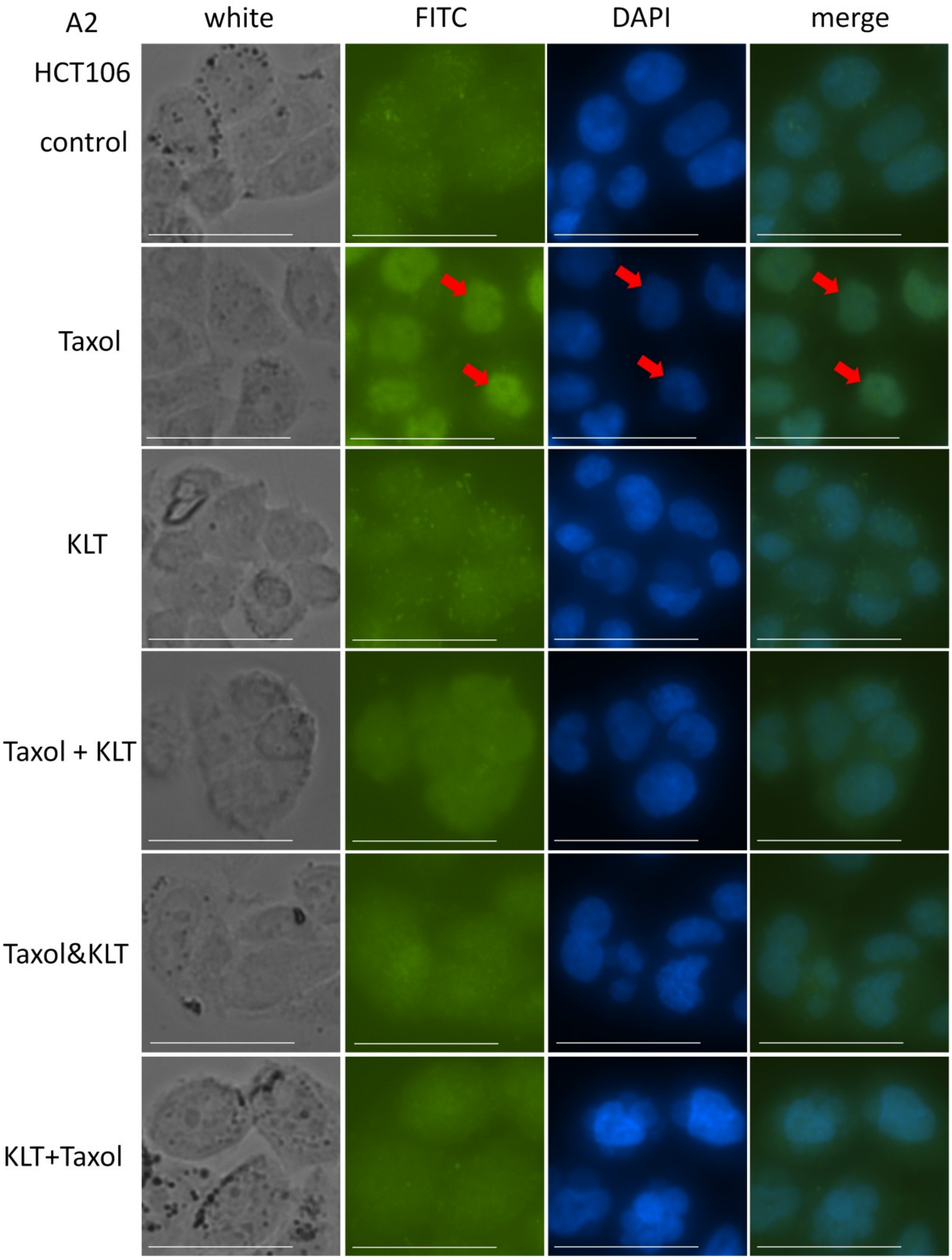
A2
white
FITC
DAPI
merge
HCT106
control
Taxol
KLT
Taxol + KLT
Taxol&KLT
KLT+Taxol

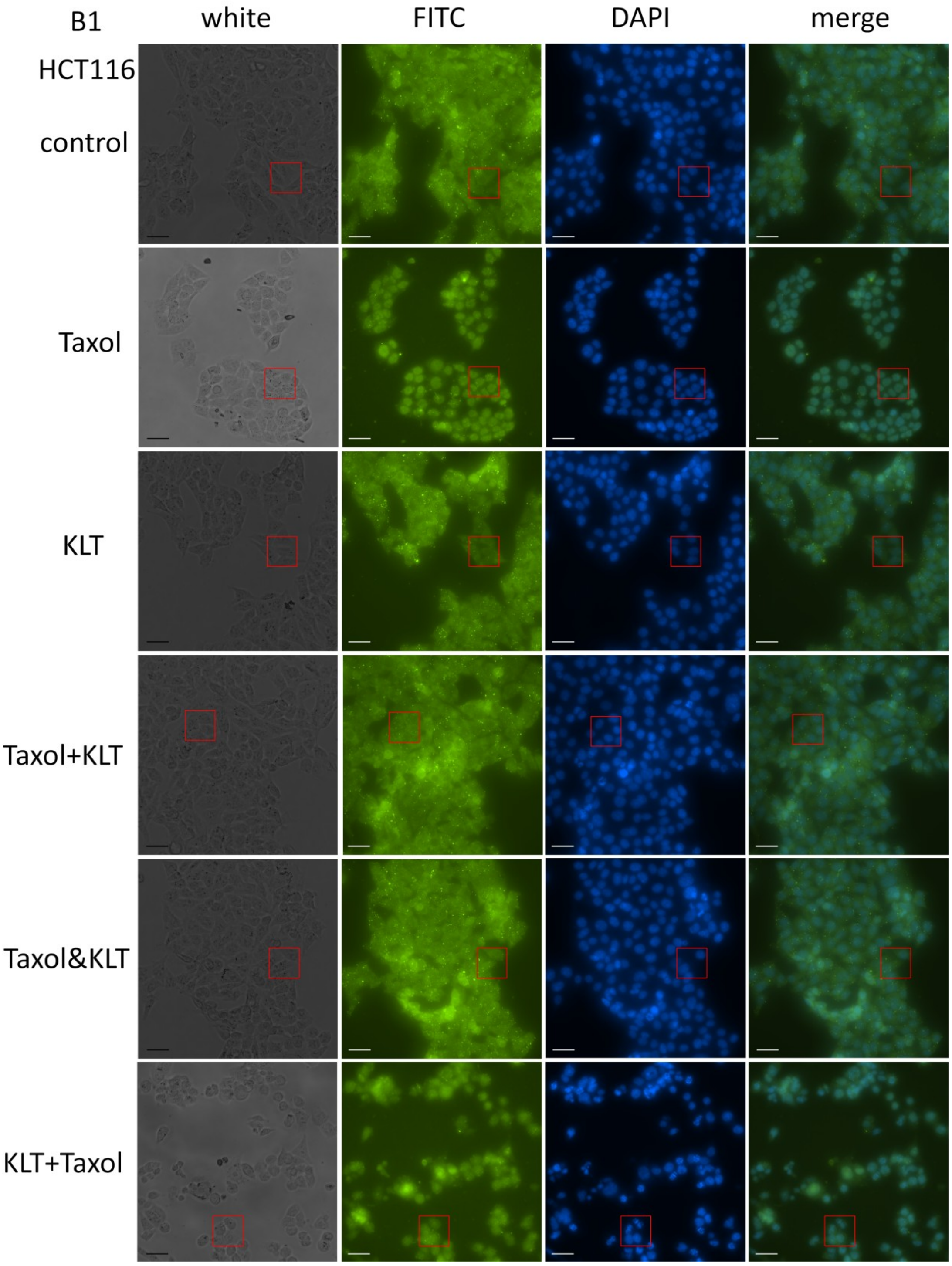
B1
white
FITC
DAPI
merge
HCT116
control
Taxol
KLT
Taxol+KLT
Taxol&KLT
KLT+Taxol

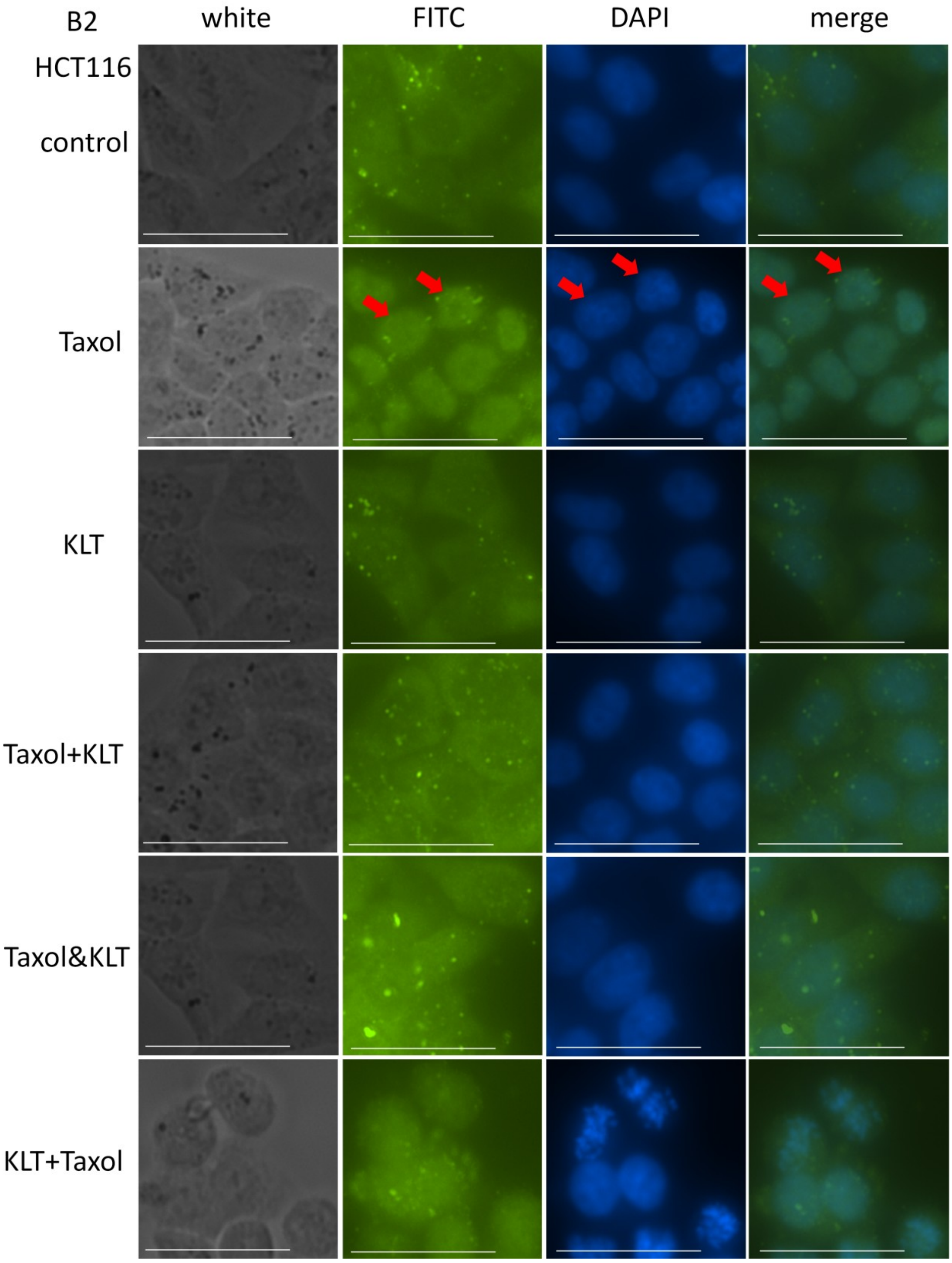
B2
white
FITC
DAPI
merge
HCT116
control
Taxol
KLT
Taxol+KLT
Taxol&KLT
KLT+Taxol

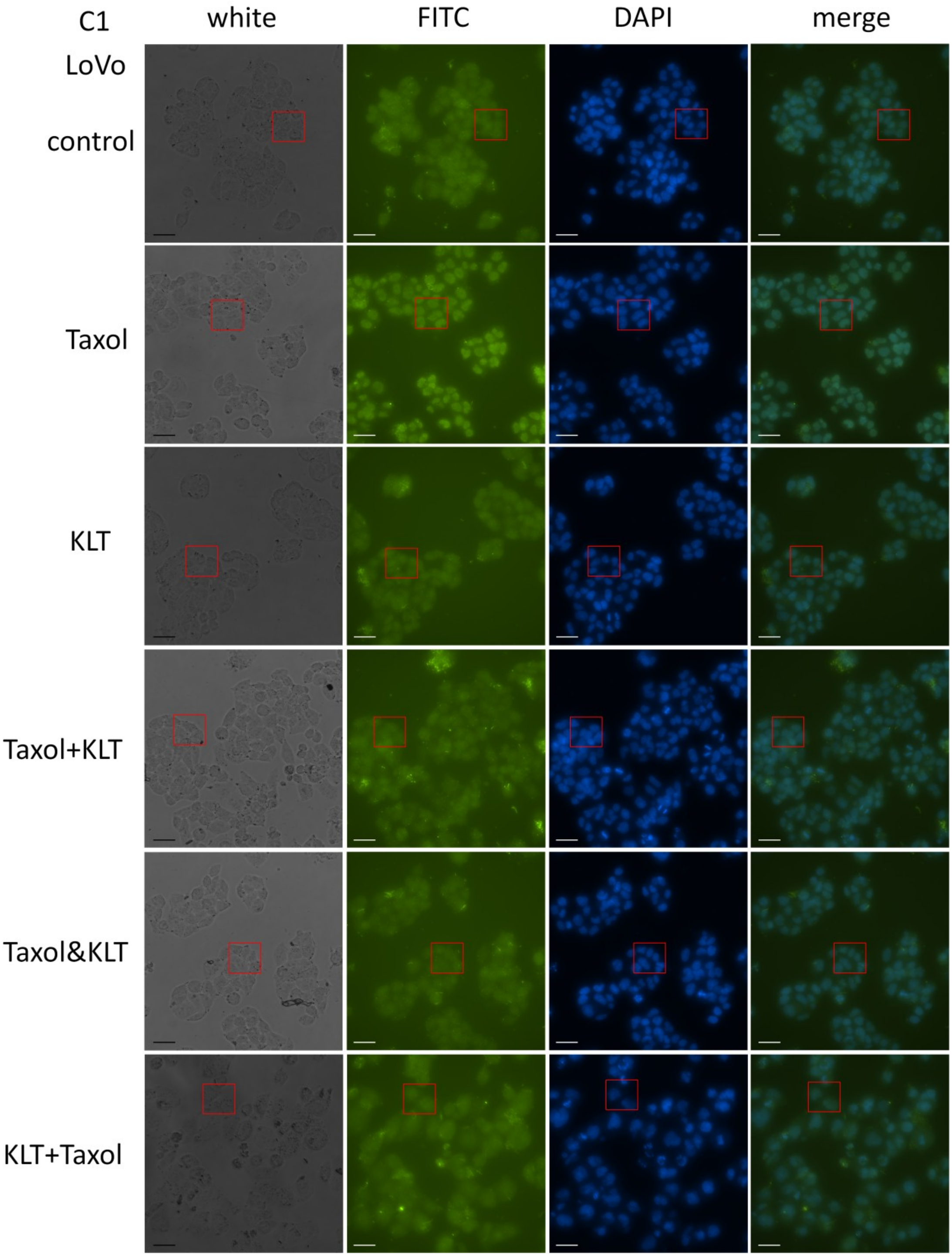
C1
white
FITC
DAPI
merge
LoVo
control
Taxol
KLT
Taxol+KLT
Taxol&KLT
KLT+Taxol

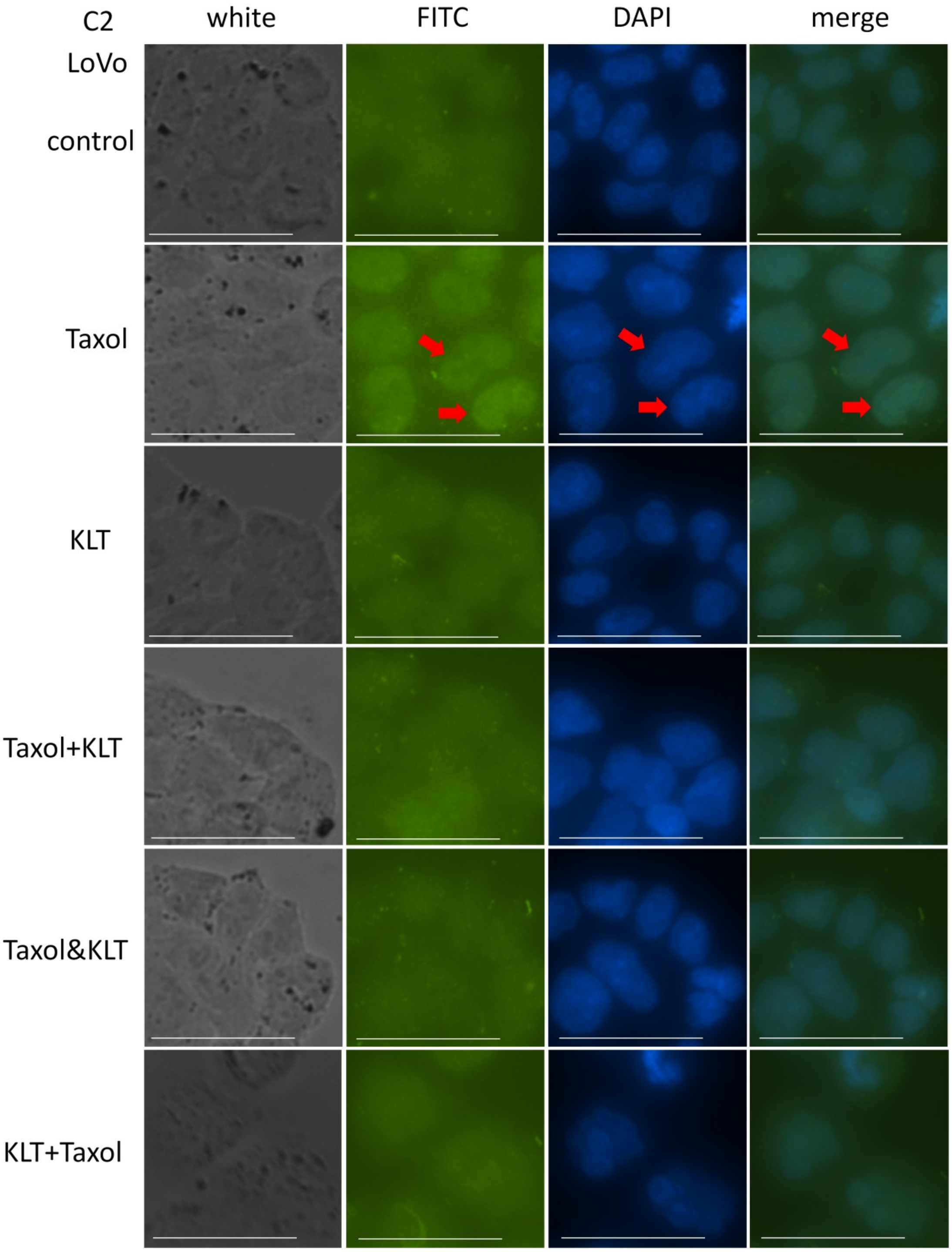

C2
white
FITC
DAPI
merge
LoVo
control
Taxol
KLT
Taxol+KLT
Taxol&KLT
KLT+Taxol

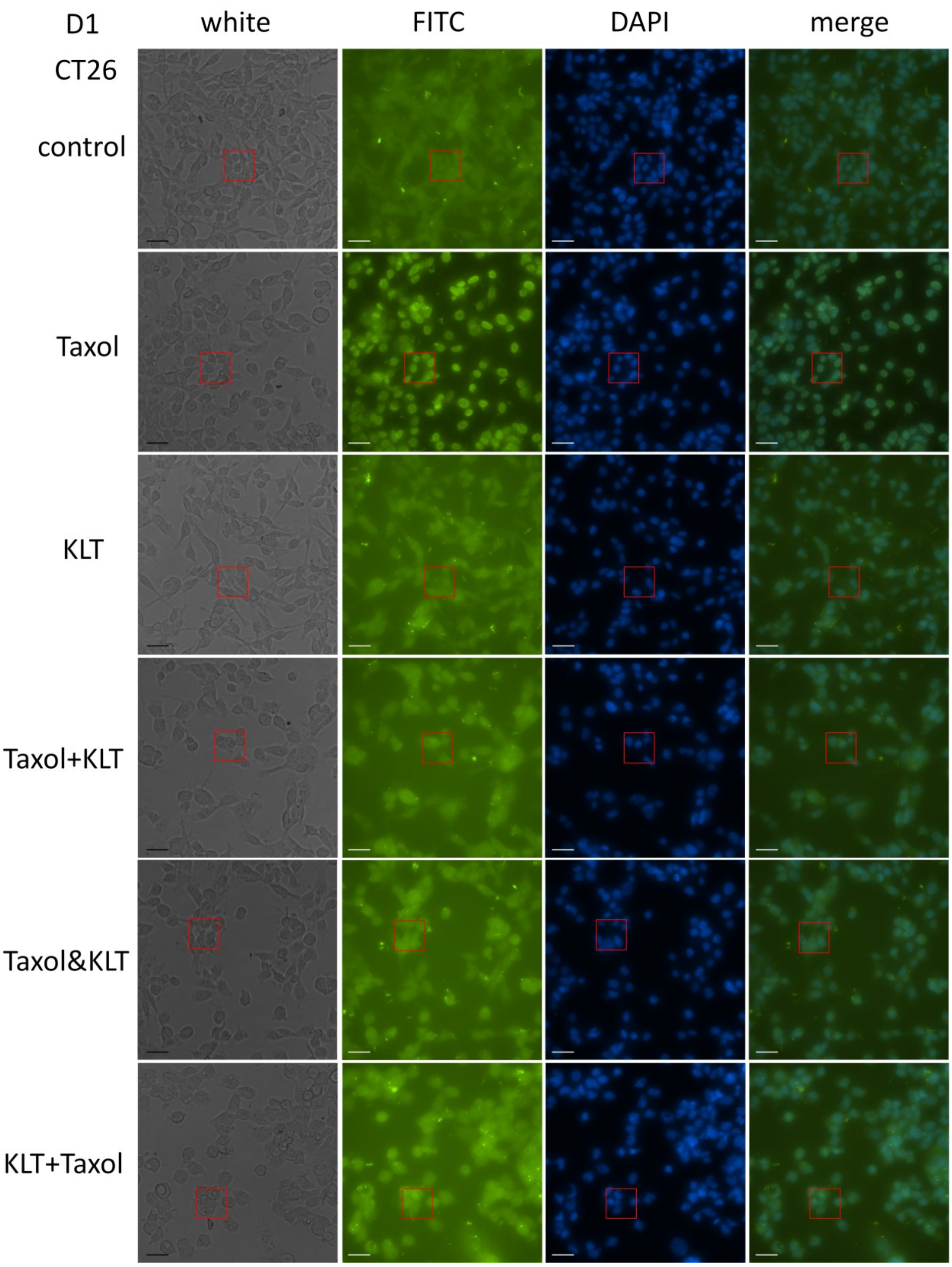
D1
white
FITC
DAPI
merge
CT26
control
Taxol
KLT
Taxol+KLT
Taxol&KLT
KLT+Taxol

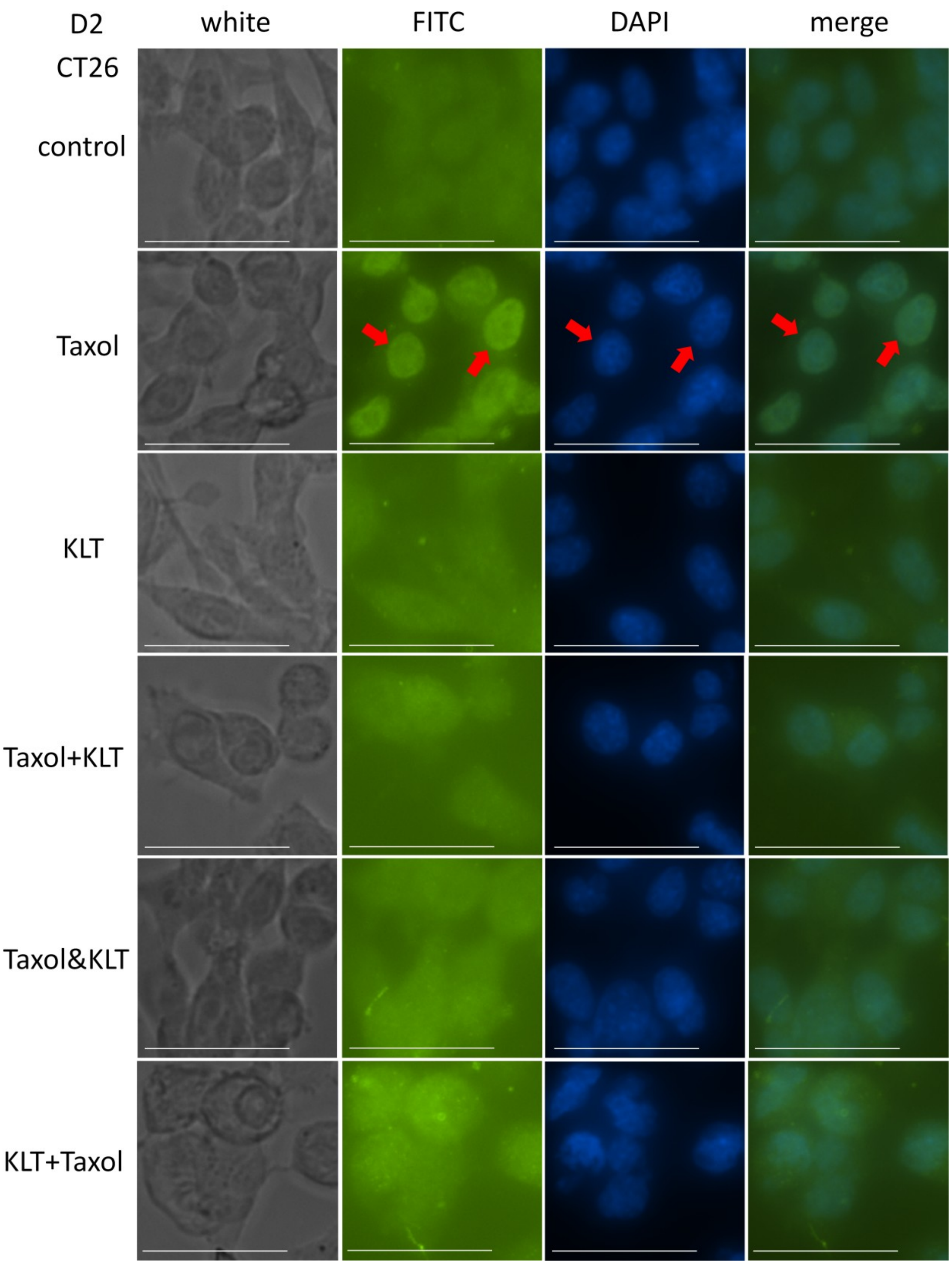

Fig.S2. Fluorescent micrographs of four colorectal cancer cell lines immunostained with NF-κB antibody followed by FITC-conjugated antibody. The drug treatment groups are listed in the left column and are described in “Kanglaite and Taxol treatment”. Activated NF-κB is highly expressed in the nuclei by single Taxol treatment, but the combinations containing KLT treatment have almost no activated NF-κB expression in the nuclei. The scale bars represent 20 μm. All images in red box are enlarged in order to determine whether NF-κB is translocated to nuclear. Red arrows indicate that NF-κB has high expression level in nuclear by comparison of FITC and DAPI stain. (A1) HCT106; (A2) Enlarged images of red box in A1; (B1) HCT116; (B2) Enlarged images of red box in B1; (C1) LoVo; (C2) Enlarged images of red box in C1; (D1) CT26; (D2) Enlarged images of red box in D1.

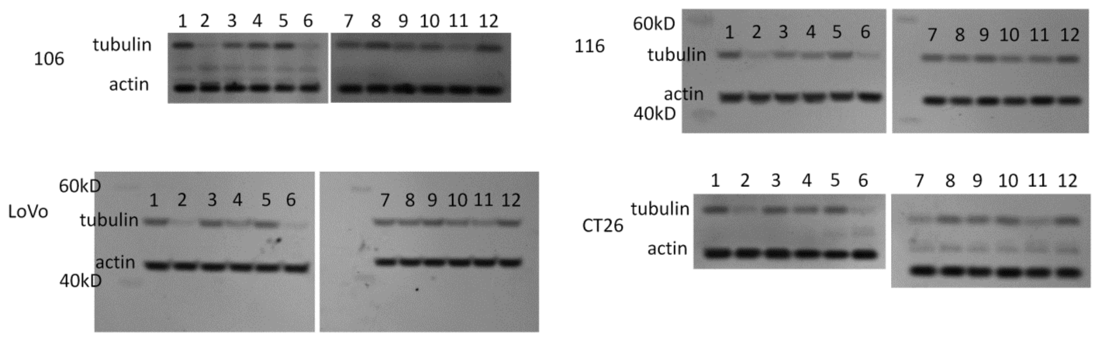

Fig.S3. Full length blots of Fig.2: Western blotting analysis of α-tubulin polymerization in four colorectal cancer cell lines. The number above images represents: 1: control-S; 2: control-P; 3: Taxol-S; 4: Taxol-P; 5: KLT-S; 6: KLT-P; 7: T+K-S; 8: T+K-P; 9: T&K-S; 10: T&K-P; 11: K+T-S; 12:K+T-P. Where "S" represents soluble tubulin and "P" represents polymerized tubulin.

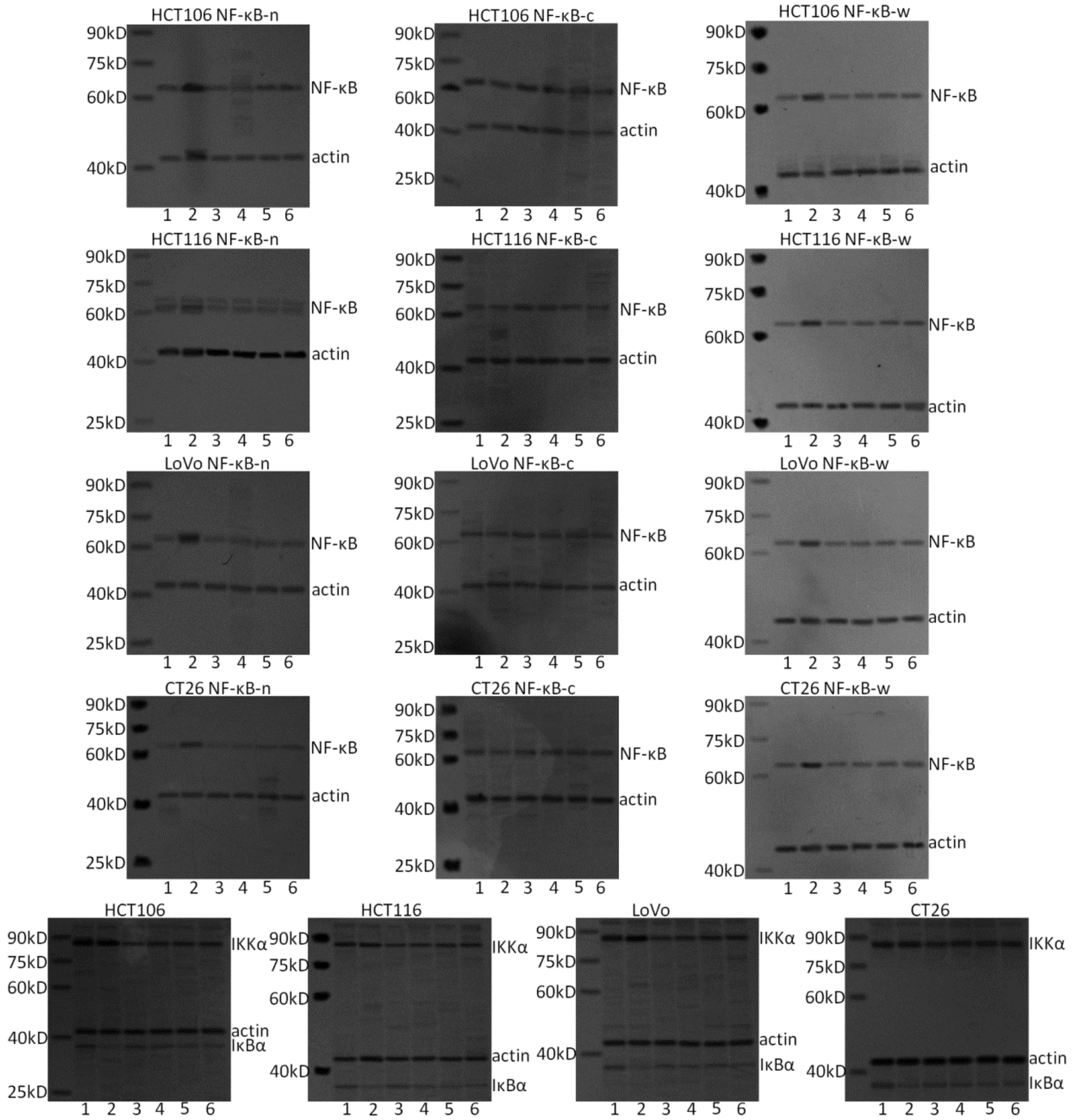

Fig.S4: Full length blots of Fig.3, which include western blotting analysis of NF-κB, IKKα and IκBα expression in four colorectal cancer cell lines, HCT106, HCT116, LoVo, and CT26. The numbers represent different treatment groups: 1: control; 2: Taxol; 3: KLT; 4: Taxol+KLT; 5: Taxol&KLT; and 6: KLT+Taxol.

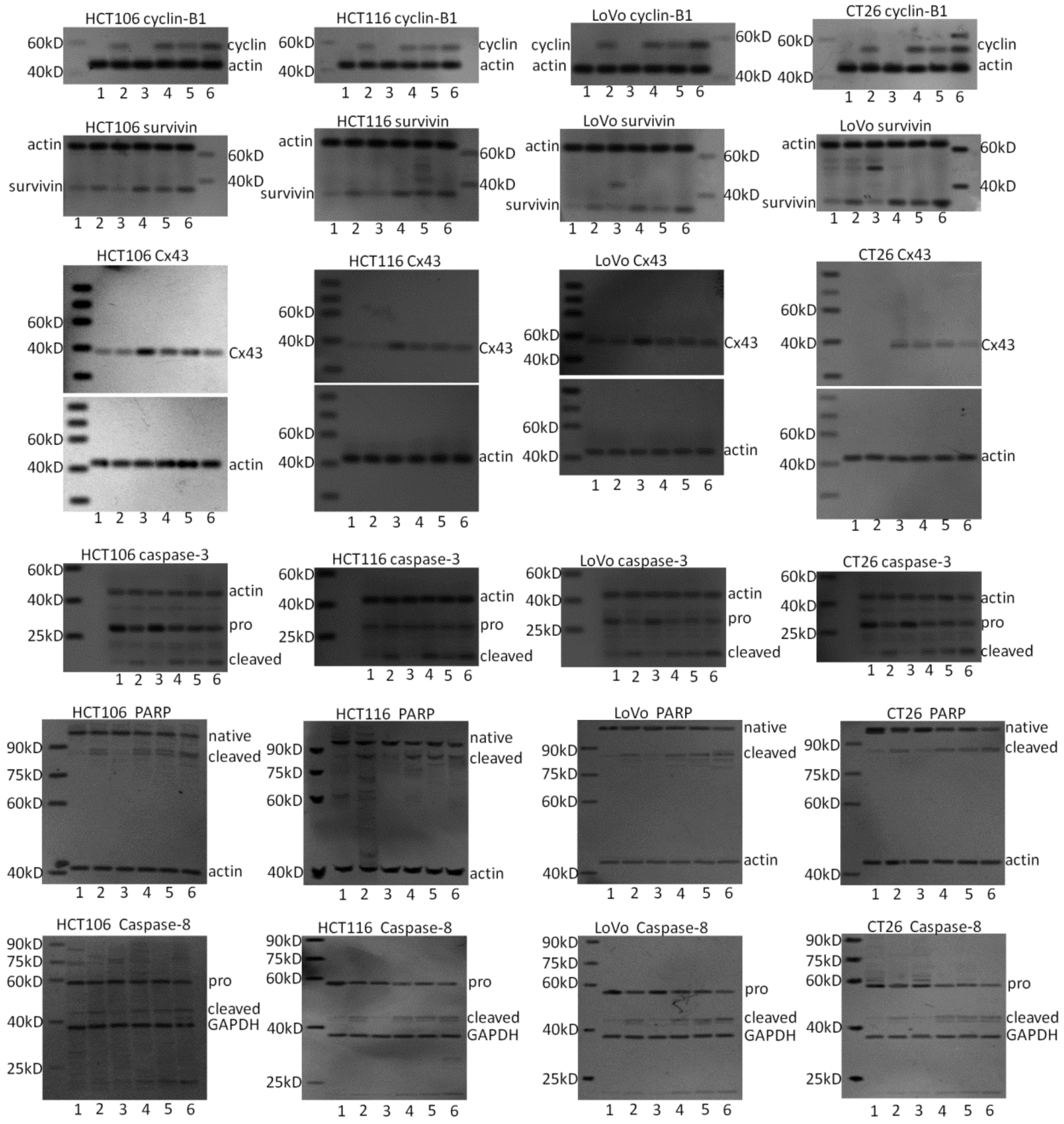

Fig.S5: Full length blots of Fig.4, which include western blotting analysis of cyclin B1, survivin, Cx43, caspase-3, PARP and caspase-8 expression in four colorectal cancer cell lines, HCT106, HCT116, LoVo, and CT26. The numbers represent different treatment groups: 1: control; 2: Taxol; 3: KLT; 4: Taxol+KLT; 5: Taxol&KLT; and 6: KLT+Taxol.
